# EDB-FN Targeted Peptide–Drug Conjugates for Use against Prostate Cancer

**DOI:** 10.3390/ijms20133291

**Published:** 2019-07-04

**Authors:** Shang Eun Park, Kiumars Shamloo, Timothy A. Kristedja, Shaban Darwish, Marco Bisoffi, Keykavous Parang, Rakesh Kumar Tiwari

**Affiliations:** 1 Center for Targeted Drug Delivery, Department of Biomedical and Pharmaceutical Sciences, Chapman University School of Pharmacy, Harry and Diane Rinker Health Science Campus, 9401 Jeronimo Road, Irvine, CA 92618, USA; 2 Biochemistry and Molecular Biology, Schmid College of Science and Technology, Chapman University, Orange, CA 92866, USA; 3 Organometallic and Organometalloid Chemistry Department, Chemical Industries Research Division, National Research Centre, 33 EL Bohouth St. (former EL Tahrir st.) Dokki, Giza 12622, Egypt

**Keywords:** antiproliferative assay, conjugation, docetaxel, doxorubicin, extra domain B, fibronectin, Fmoc/tBu, peptide–drug conjugate, prostate cancer, solid-phase synthesis, targeting

## Abstract

Prostate cancer (PCa) is the most common malignancy in men and is the leading cause of cancer-related male mortality. A disulfide cyclic peptide ligand [CTVRTSADC] **1** has been previously found to target extra domain B of fibronectin (EDB-FN) in the extracellular matrix that can differentiate aggressive PCa from benign prostatic hyperplasia. We synthesized and optimized the stability of ligand **1** by amide cyclization to obtain [KTVRTSADE] **8** using Fmoc/tBu solid-phase chemistry. Optimized targeting ligand **8** was found to be stable in phosphate buffered saline (PBS, pH 6.5, 7.0, and 7.5) and under redox conditions, with a half-life longer than 8 h. Confocal microscopy studies demonstrated increased binding of ligand **8** to EDB-FN compared to ligand **1**. Therefore, we hypothesized that the EDB-FN targeted peptides (**1** and **8**) conjugated with an anticancer drug via a hydrolyzable linker would provide selective cytotoxicity to the cancer cells. To test our hypothesis, we selected both the normal prostate cell line, RWPE-1, and the cancerous prostate cell lines, PC3, DU-145, LNCaP, and C4-2, to evaluate the anticancer activity of synthesized peptide–drug conjugates. Docetaxel (Doce) and doxorubicin (Dox) were used as anticancer drugs. Dox conjugate **13** containing disulfide linkage showed comparable cytotoxicity versus Dox after 72 h incubation in all the cancer cell lines, whereas it was found to be less cytotoxic on RWPE-1, suggesting that it can act as a Dox prodrug. Doce conjugate **14** was found to be less cytotoxic in all the cell lines as compared to drug alone.

## 1. Introduction

Prostate cancer (PCa) is the most common form of cancer in males and the second leading cause of death after lung cancer in the United States. According to the American Cancer Society, it is also the first leading cause of cancer mortality in males aged 65 and older in the United States [1]. There are several treatment options for prostate cancer patients, which depend on the clinical risk assessment at the time of diagnosis, including tumor stage and grade, but also patient age and health. High-risk local disease is routinely addressed by local treatment with curative intent, such as radical prostatectomy followed by adjuvant radiation or androgen ablation therapy. Unfortunately, a fraction of patients (~20%) will progress to more aggressive disease, manifested by a metastatic spread of primary tumor cells, which in the United States results in a mortality rate of ~30,000 individuals per year [2]. Recurrent systemic disease, including metastatic castration resistance prostate cancer (mCRPC) after androgen ablation therapy, is typically treated with chemotherapy, most notably with docetaxel (Doce) and cabazitaxel [3,4]. The anthracycline doxorubicin (Dox) as a main chemotherapeutic drug for prostate cancer remains in the exploratory setting, while its synthetic chemical modifications, such as the anthracenedione (mitoxantrone) is in clinical use [5]. Regardless of the chemotherapeutic drug in use, whether in single or in combinatorial regimens, the major problem of off-target effects leads to substantial morbidity due to side effects. This has led to multiple lines of research into targeting strategies, including the use of liposomes and nanoparticles [6,7]. We have previously reported the synthesis of cell-penetrating peptide–drug conjugates for the delivery of anticancer drugs [8]. Targeted therapy could deliver the cytotoxic or therapeutic molecules to cancer cells, thereby minimizing exposure to normal cells. This promises to reduce the side effects associated with drug-based systemic therapies [9]. The use of tumor-specific or homing peptides as an example of targeted delivery in cancer was shown by Ruoslahti et al. [10]. A heightened efficiency of the delivery of a cytotoxic agent may also counteract the efflux of drugs due to multidrug resistance pumps. Hence, tumor targeting with enhanced selectivity should be more beneficial for an effective therapeutic outcome. Several ligands have been reported for targeting cytotoxic or chemotherapeutics molecules, such as monoclonal antibodies, peptide hormones, receptor-binding ligands, aptamers, oligosaccharides, vitamins, folic acids, nanoparticles, and DUPA [11]. However, most of these targeting ligands lack specificity for tumor cells, which in the clinical setting, is expected to lead to side effects due to their effect on non-malignant normal cells. Therefore, there is a need to develop more tumor-specific targeting modalities. 

**Figure 1:**
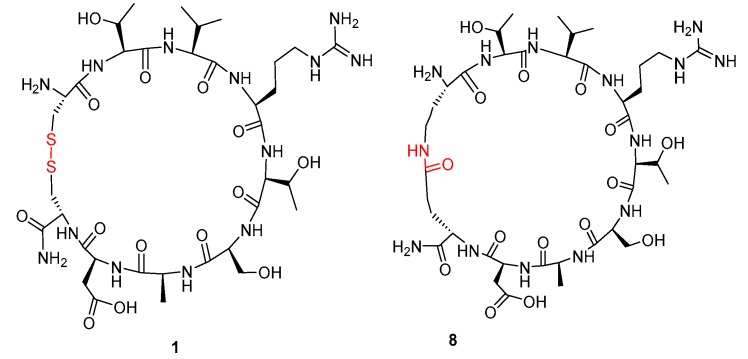
Chemical structures of peptide ligands **1** and **8**.

The extracellular matrix (ECM) is a microenvironment within all types of tissues and organs containing non-cellular components such as collagen, fibroblasts (which ARE cells), fibronectins (FNs), and blood vessels as a part of the basal lamina for the support of epithelial cells [12]. The ECM is responsible for adhesion, migration, cell-to-cell communication, homeostasis, growth, and proliferation of the cells, and thus contributes significantly to metastasis of tumors [13]. In malignant tumors, the microenvironment exhibits unusual overexpression of cancer-related proteins, such as the extra-domain B fibronectin (EDB-FN). EDB-FN is a high-molecular glycoprotein that mediates cell adhesion and migration [14]. EDB-FN has been reported to be found around new blood vessels of tumors and provides a promising specific biomarker for cancer [15,16,17]. Therefore, EDB-FN could be used for targeted therapy in PCa. For example, Zheng et al. showed the use of a specific peptide ligand [CTVRTSADC] **1**, which targets EDB-FN in PCa (Figure 1). This ligand was used in the diagnosis of later stages of PCa, differentiating them from benign hyperplasia. Later, ligand **1** was used as a targeted contrast agent in MRI application for PCa [18]. Therefore, peptide ligand **1** could be selected as a potential targeting ligand to develop targeted chemotherapeutics for PCa.

The rationale for the selection of peptides as targeting ligands is due to the fact that peptides are highly selective, safer, and tolerable agents in vivo. The specificity of the peptide ligand for its target could be used to deliver an anticancer drug to their target in cancer. Thus, we hypothesized that the conjugates of EDB-FN targeted peptide ligand with anticancer drugs containing a suitable linker will release the anticancer drug in the PCa and minimize side effects in normal cells. Here in this study, we report the synthesis and modification in peptide ligand **1**, developing peptide-anticancer drug conjugates, their characterization, stability, and in vitro assays against a panel of PCa and normal prostate cell lines. 

## 2. Results and Discussion

### 2.1. Chemistry

We synthesized two different cyclic peptide EDB-FN targeting [CTVRTSADC] **1** and [KTVRTSADE] **8** (Figure 1) using solid phase peptide chemistry and used for the conjugation with anticancer drugs. Two anticancer drugs, Dox and Doce, were used for the conjugation. We adopted six carbon long aminohexanoic acid to provide spacer followed by cathepsin-B enzyme sensitive linker to release the drug moiety efficiently. Two types of dipeptide cathepsin-B enzyme sensitive linkers including valine and citrulline (VCit) and phenylalanine and lysine (FK) were adopted in the early phase of the study, but later a tetrapeptide linker with better release profile involving glycine, phenylalanine, leucine, and glycine (GFLG) was chosen. To facilitate drug attachment, hydrazone, disulfide, and thiomaleimide linkages were used. To provide a spacer for better recognition of the cathepsin-B sensitive linker, dipeptide of glycine was attached on hydrazone linkage, or additional cysteine was attached on the *N*-terminal to facilitate disulfide linkage. 

#### 2.1.1. Synthesis of Targeting Ligand

**Scheme 1:**
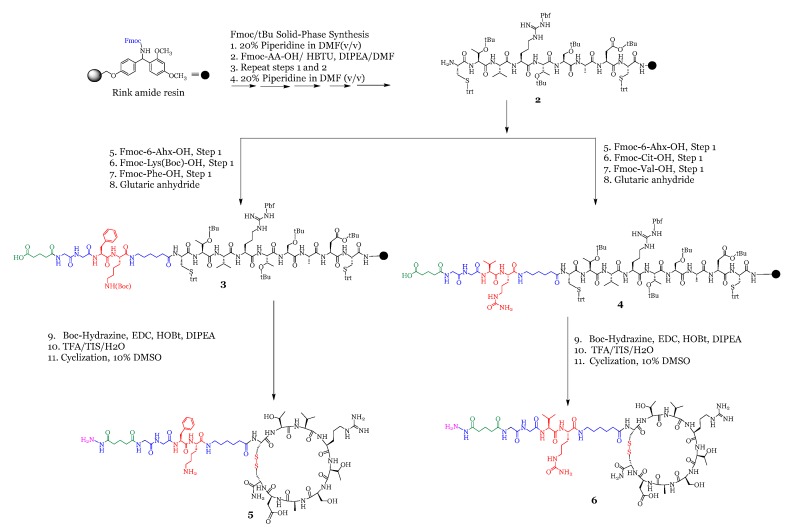
Synthesis of hydrazine-glutarate-GG-FK-C_6_-[CTVRTSADC] **5** and hydrazine-glutarate-GG-VCit-C_6_-[CTVRTSADC] **6**.

Selected peptide ligand [CTVRTSADC] **1** contained a disulfide bridge between *C*- and *N*-terminal cysteine residues and was generated after the first-generation screening of phage display library bound to EDB-FN as previously reported [15]. Using this peptide, we designed the peptide-linker hydrazine-glutarate-GG-FK-C_6_-[CTVRTSADC] **5** and hydrazine-glutarate-GG-VCit-C_6_-[CTVRTSADC] **6** containing a cathepsin-sensitive linker (Scheme 1). 

This disulfide linkage in targeting peptide **1** may be hydrolyzed in vivo due to the presence of glutathione or under physiological condition, which may reduce targeting efficiency of the ligand. Alternatively, we designed a more stable analog of ligand **1**, which contains lactam cyclization between lysine and glutamic side chain in place of cysteine amino acids. Therefore, two cyclic peptides [CTVRTSADC] **1** and [KTVRTSADE] **8** were synthesized using standard Fmoc/tBu solid-phase peptide synthesis (SPPS) on Rink amide resin as solid support. All the peptides were purified by preparative Reverse-Phase High-Performance Liquid Chromatography (RP-HPLC) and analyzed by Matrix-Assisted Laser Desorption/Ionization-Time of Flight (MALDI-TOF) mass spectroscopy (see supporting material). Most of the peptides showed an [M + H]^+^ peak in the spectra, which is due to the presence of the protonated amino group. We also observed mass fragments presumably because of the breakdown of disulfide linkage to thiol (SH) groups when higher laser energy was used in the MALDI-TOF mass spectroscopy. 

#### 2.1.2. Synthesis of Peptide-Linker Conjugates

A spacer of six carbon (C_6_) was included using Fmoc-aminohexanoic acid (Fmoc-Ahx-OH) in the design of peptide–drug conjugates to allow targeting ligand to recognize their binding motif. The release of drug from the conjugates was expected using a suitable linker. A cathepsin enzyme based linker was selected to allow the drug to be released at the site of the tumor [19]. Cathepsin-based linkers are dipeptides (FK, VCit) or tetrapeptides (GFLG), which release drug moiety efficiently from the conjugate as previously reported [20,21,22].

Scheme 1 depicts the synthesis of various cathepsin linkers in the peptide **1** cyclized through a disulfide linkage. A spacer of two glycine amino acids was added in the peptide sequence to avoid any steric hindrance for enzyme recognition of the linker. Furthermore, a hydrazine group was also included at the *N*-terminal to allow the selected model anticancer drug (Dox) to be conjugated using hydrazone linkage. 

**Scheme 2:**
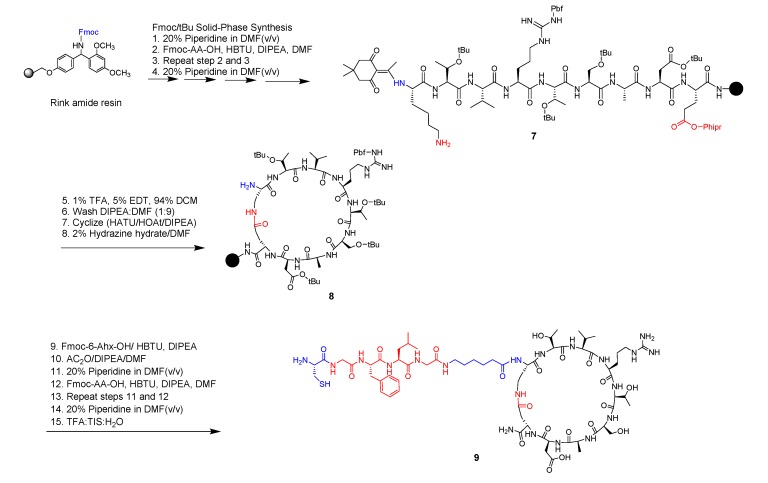
Selected synthesis of C-GFLG-C_6_-[KTVRTSADE] **9** containing lactam cyclization.

Modified peptide ligand (KTVRTSADE) **8** was assembled on the resin with the modification of reported methodology of ligand **1** [18]. Scheme 2 depicts the synthesis of **8** that has lysine and glutamic acid residues instead of cysteine residues. Peptidyl resin went through lactam cyclization and conjugated with a linker. The orthogonal protected groups used during the synthesis for lysine and glutamic acid were 1-(4,4-dimethyl-2,6-dioxocyclohex-1-ylidene)ethyl (Dde) and 2-phenylisopropyl (Phipr), respectively. The protected linear version of peptide **8** was assembled on Rink amide resin followed by selective deprotection of Fmoc and Phipr group in the side chains. Lactam cyclization afforded side-chain cyclized intermediate peptide. After cleavage of a part of the resin, the calculated mass of **8** was 986.5145 [M]^+^ that was consistent with experimental value 987.2699 [M + H]^+^. Scheme 2 shows the synthesis of the linker, spacer, and targeting moiety in peptide **9**. Cysteine residue was incorporated at the *N*-terminal for functionalization in drug conjugation.

#### 2.1.3. Conjugation of Anticancer Drugs with Peptide-Linker Conjugates

**Scheme 3:**
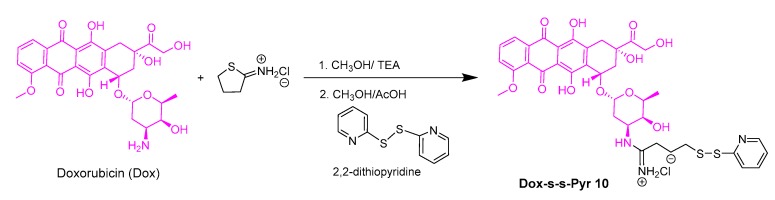
Synthesis of Dox-disulfide-pyridyl **10**.

**Scheme 4:**
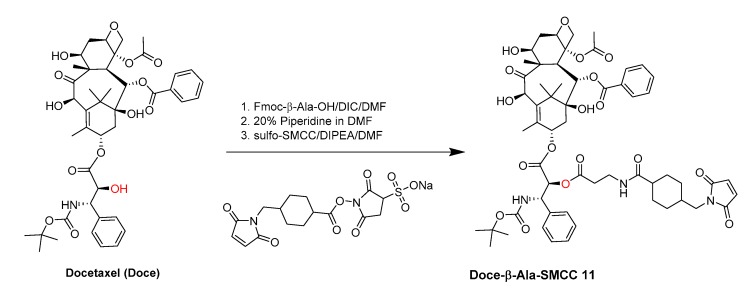
Synthesis of Doce-βA-SMCC **11**.

**Scheme 5:**
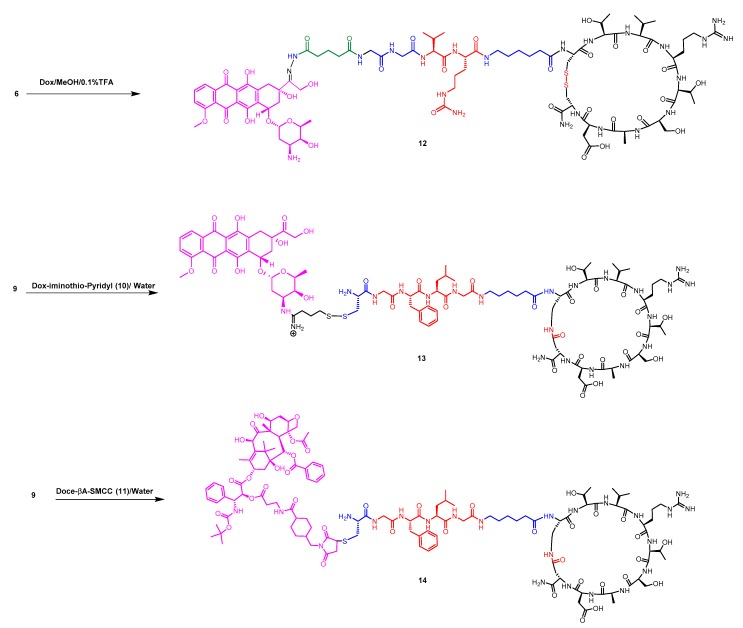
Conjugation of Dox, Dox-SH, and Doce-βA-SMCC with the peptide targeting moiety through a hydrolyzable linker

Doxorubicin (Dox) and docetaxel (Doce) were selected as model anticancer drugs for the conjugation with the synthesized linker-EDB peptides to form compound Dox-s-s-GFLG-C_6_-[KTVRTSADE] **13** and Doce-βA-thioether-CGFLG-C_6_-[KTVRTSADE] **14**. Dox and Doce have been widely used in chemotherapy against several cancers, including PCa [23]. The intermediate compound Dox-disulfide-pyridine was prepared by reaction of Dox with 2-iminothiolane followed by activation of the sulfhydryl group by reacting with dithiodipyridine at the room temperature (Scheme 3). Similarly, Doce was functionalized with Fmoc-β-alanine-OH to reduce the steric hindrance in the reaction with the linker-EDB peptide. The C2’ hydroxyl group in the Doce has been previously reported to be more reactive as compared to other hydroxyl groups in the compound. The other hydroxyl groups have shown to have low chemical reactivity due to the presence of more steric hindrance [24,25,26]. After Fmoc removal, the crude esterified Doce-β-alanine was purified using RP-HPLC. The purified compound showed one major peak with expected mass and was concentrated to use for the next reaction. A bifunctional linker sulfosuccinimidyl 4-(*N*-maleimidomethyl)cyclohexane-1-carboxylate) (sulfo-SMCC) was used to facilitate conjugation of Doce-β-alanine-NH_2_ to form Doce-β-alanine-SMCC **11**, which was utilized in the conjugation with peptide **9** (Scheme 4). In another strategy, ketone group at the C13 position in the Dox was reacted under mildly acidic condition with the hydrazine group present at the EDB peptide-linker **6** to form hydrazone linkage in conjugate **12** as shown in Scheme 5.

#### 2.1.4. Conjugation of Anticancer Drugs with Peptides

Three peptide–drug conjugated compounds (**12**, **13**, and **14**) were synthesized (Scheme 5). In the first reaction, Dox was reacted under a mild acidic condition with hydrazine-glutarate-GG-VCit-C_6_-[CTVRTSADC] **6** for 20 h at room temperature to afford the Dox–hydrazone peptide conjugate **12** (Scheme 5). In the second attempt, Dox-activated disulfide compound **10** was reacted with C-GFLG-C_6_-[KTVRTSADE] **9** to afford Dox-s-s-C-GFLG-C_6_-[KTVRTSADE] **13**. In the third attempt, the Doce-*β-*alanine-SMCC **11** was reacted with sulfhydryl group in **9** using phosphate buffered saline (PBS, pH 8.0) containing 20 mM EDTA for 17 h to form Doce-thioether-CGFLG-C_6_-[KTVRTSADE] **13**. All the conjugates were purified and characterized using RP-HPLC and MALDI-TOF mass spectrometry, respectively. Table 1 shows the chemical analysis and details of synthesized peptides and peptide–drug conjugates.

#### 2.1.5. Conjugation of 5(6)-Carboxyfluorescein (FAM) Motif with Peptides

**Scheme 6:**
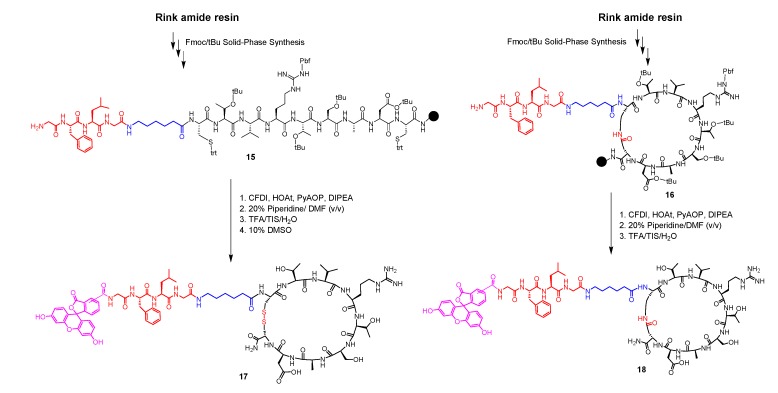
Conjugation of FAM with the peptide targeting moiety through a hydrolyzable linker.

Peptides **15** and **16** containing linker and targeting ligand (**1** and **8**, respectively) were conjugated with a carboxyfluorescein dye to determine whether ligand **8** showed higher localization and targeting toward EDB-FN as compared to its disulfide cyclized counterpart **1**. A 5(6)-carboxyfluorescein diisobutyrate (CFDI) was coupled to *N*-terminus of glycine in both peptides (**15** and **16**) in the presence of PyAOP, HOAt, and DIPEA using anhydrous DMF. Fluorescent-tagged peptides (**17** and **18**) were purified and characterized using RP-HPLC and MALDI mass spectrometry, respectively (Scheme 6).

#### 2.1.6. Stability and Hydrolysis

**Figure 2:**
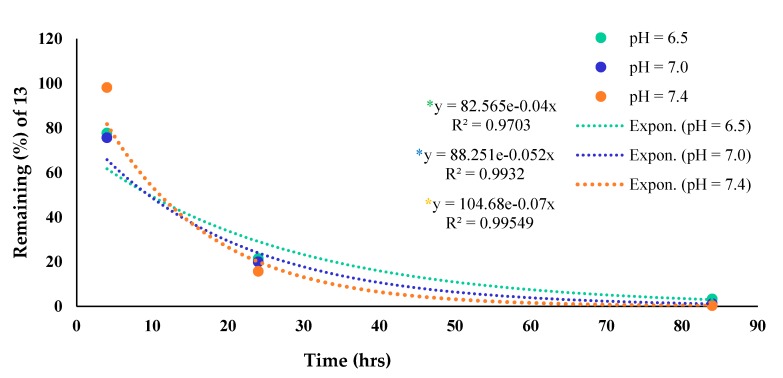
Conjugate **13** in PBS at different pH. The percent remaining of conjugate **13** was quantified using the area under the curve (AUC) in analytical HPLC.

**Figure 3:**
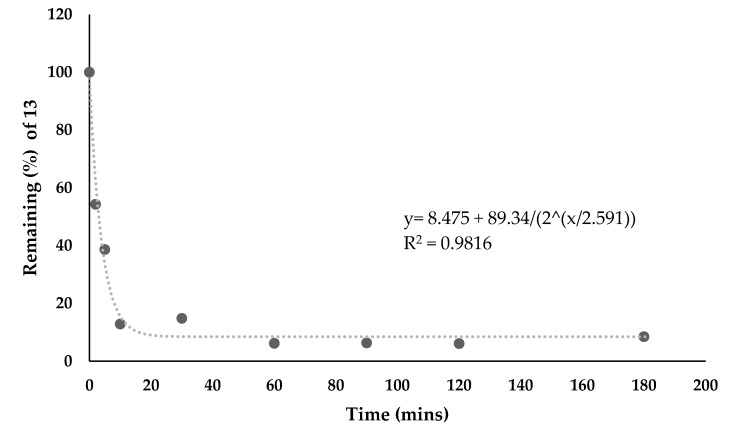
Stability analysis of conjugate **13** under reducing conditions using dithiothreitol (DTT). The experiment was done in PBS at pH 7.4. The percent remaining was quantified using the AUC, and the trendline equation gives the half-life of 2.6 min with an R^2^ of 0.98. Fitting was done online using MyCurveFit.

**Figure 4:**
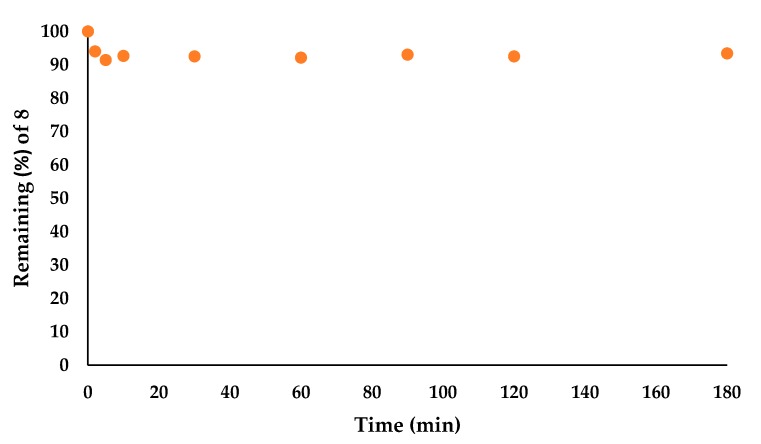
Stability of peptide **8** ([KTVRTSADE]) in pH 7.4 PBS with DTT. The percent remaining was calculated from the AUC.

**Figure 5:**
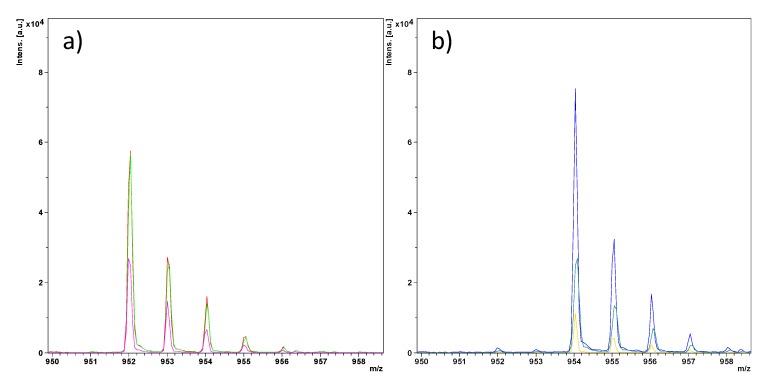
Peptide **1** before (**a**) and after 5 min (**b**) incubation with DTT. The higher molecular weight shows a full reduction of peptide **1** to the linear counterpart of (CTVRTSADC) within 5 min.

The stability of selected synthesized doxorubicin-peptide conjugate **13** was evaluated under different physiological conditions to mimic the in vivo environment, such as different pH, under redox condition, and in the human serum as depicted in the Figure 1, Figure 2, Figure 3 and Figure 4. Conjugate **13** showed high stability in PBS with a half-life of ~10 h in pH values ranging from 6.5 to 7.4 (Figure 2). It also appears that the dox peptide conjugate **13** is stable at pH tested from 6.5 to 7.4. No drastic differences observed between stability in three different physiological pHs. Furthermore, conjugate **13** were treated with redox condition using dithiothreitol (DTT) to confirm the release of Dox from conjugate **13** that allows the free drug becomes bioactive. Figure 3 shows the hydrolysis of conjugate **13** within 2.6 min when incubated with 12 equivalents of DTT that confirms the release of Dox from disulfide linkage due to redox conditions. Similarly, the targeting peptides which consist of disulfide cyclization (peptide **1**) and lactam cyclization (peptide **8**) were incubated in the redox condition. In the presence of DTT, the targeting peptide ligand **1** behaved as expected and released reduced disulfide linkage quickly. During HPLC analysis of the ligand, it was found to be too difficult to separate the linear and cyclic forms of CTVRTSADC, so MALDI mass spectrometry was used to obtain a qualitative insight. Peptide **1** was reduced to its linear form that has a slightly higher mass because of the addition of two hydrogen atoms to the reduced disulfide bond into two sulfhydryl groups thereby increasing its mass by 2 Da in high-resolution MALDI-TOF mass spectroscopy (Figure 5). However, peptide **8** was stable under reducing conditions as expected (Figure 4). 

**Figure 6:**
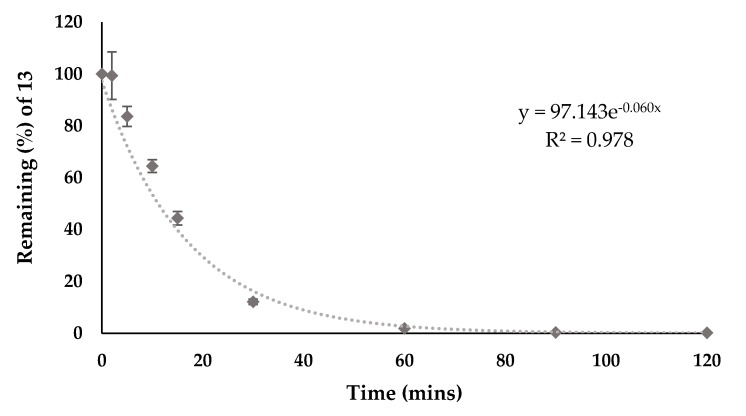
Stability of conjugate **13** (125 µM) in 25% human serum. Note the trendline gives the half-life of 11.8 min with an R^2^ of 0.978. Each point represents an average of three readings.

Dox–peptide conjugate **13** showed a quick degradation in human serum at 37 °C (Figure 6). The data shows that conjugate **13** has a half-life of approximately 11.8 min in 25% human serum. There was no visible difference in the shape of the percentage remaining vs. time plot when performed using a higher concentration of conjugate **13**. Thus, it was speculated that the rate of the reaction increased proportionally when a higher concentration (100 µM) of Dox–peptide was used. This indicates that the reaction was likely to be first-order with regard to Dox–peptide **13** under these conditions. Usually, peptides and peptide–drug conjugates have a very wide range of serum stabilities ranging from less than a min in human serum to over 200 min leading to an undetectable amount of degradation [27]. Conjugate **13** showed a half-life of 11.8 min, which displays that this peptide–drug conjugate, although does not have outstanding stability, it may have time to yield an effect on cancer cells in vivo. 

**Figure 7:**
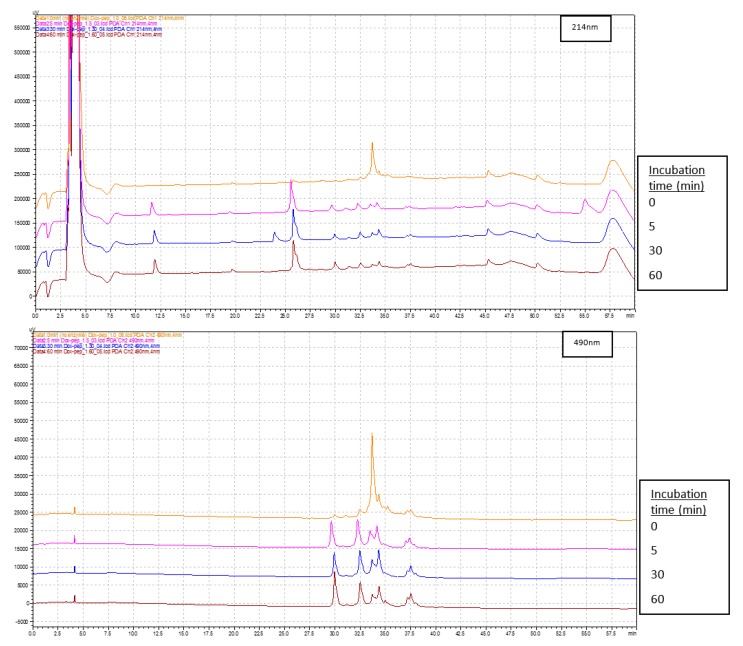
Analytical HPLC chromatogram over time for Dox-peptide conjugate **13** in human cathepsin B with absorbance detection at 214 nm (**top**) and 490 nm (**bottom**). Dox-peptide **13** retention time = 34 min. Dox alone retention time = 30 min. Note the similar shape of the 5, 30, and 60 min incubations. Also, the peak at ~30.5 min showed strong absorbance at both 214 nm and 490 nm.

Furthermore, the hydrolysis of peptide–drug conjugate **13** containing a cathepsin B cleavable linker was evaluated using in vitro assay as reported [28]. Figure 7 shows that cathepsin B enzyme recognizes GFLG linker in the conjugate **13** and cleaves it within 5 min of incubation time. Dox–peptide conjugate **13** showed a peak with the retention time of 34 min. After incubation with serum, the compound was hydrolyzed and showed different peaks. The peak with a retention time around 30.5 min was found to be dox conjugated with a couple of amino acid residues. The free doxorubicin was found with the retention time of 30 min (Supporting information, Figure S1). The peak at 30.5 min showed absorption at 490 nm and with some at 214 nm, which demonstrates amide linkage for conjugated amino acids. Therefore, the peak at 30.5 min was likely to be with Dox conjugated with a couple of amino acids. This enzymatic reaction occurred too fast to assess the kinetic nature of the reaction. However, it demonstrated the utility of the cathepsin B-sensitive linker in conjugate design.

### 2.2. Cell-Based Assays

#### 2.2.1. Peptide Cytotoxicity

To evaluate the cytotoxicity of the synthesized compounds, we selected both a normal prostate cell line (prostate epithelial cells transformed by HPV, RWPE-1) and cancerous prostate cell lines (LNCaP, PC3, DU-145, and C4-2) [29]. LNCaP is an indolent form. DU-145 is a moderatetly metastatic cell form as compared to PC3, which is an aggressive form of PCa with a high metastatic potential [30]. C4-2 cells were originally derived from LNCaP xenografts grown in castrated mice exhibiting androgen-independence and were found to have elevated expression of EDB-FN, which would help explore the targeting of peptide conjugate [31]. Evaluation of the cytotoxicity of synthesized targeting peptides hydrazine-glutarate-GG-FK-C_6_-[CTVRTSADC] **5** and hydrazine-glutarate-GG–VCit–C_6_-[CTVRTSADC] **6** as compared to peptide C-GFLG-C_6_-[KTVRTSADE] **9** were performed on different timelines (24, 48, and 72 h) using a panel of cell lines such as RWPE-1, LNCaP, PC3, DU-145, and C4-2 as depicted in Figure S2. These peptides were used in the conjugation of anticancer drugs connecting through three different types of peptidic cathepsin linkers such as phenylalanine lysine (FK), valine citrulline (VCit), and glycine phenylalanine leucine glycine (GFLG). These peptides were used to perform a cytotoxicity assay at a selected concentration of 5 µM as used previously in screening peptide–drug conjugates in different cancer cell lines [8]. Figure S2 (supporting information) showed that peptides **5, 6, 9** did not show any significant cytotoxicity to normal human prostate epithelial cells (RWPE-1), and cancerous prostate cell lines LNCaP, C4-2, PC3, and DU-145 after 72 h. These results are not surprising since the targeting peptides are not expected to have any intrinsic antiproliferative activity.

**Figure 8:**
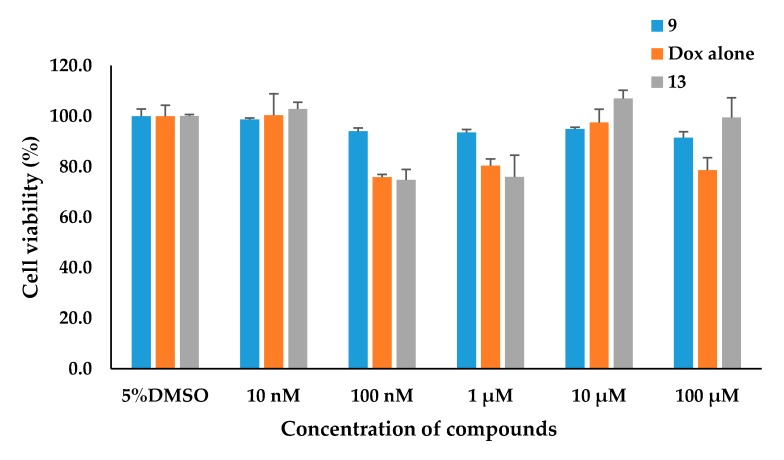
Cytotoxicity of peptide **9**, conjugate **13** (Dox-s-s-CGFLG-C6-[KTVRTSADE]) and Dox on a PC3 prostate cancer cell line after two hours of incubation at various concentrations.

The determination of cytotoxicity of peptide C-GFLG-C_6_-[KTVRTSADE] **9** and selected peptide–drug conjugate Dox-s-s-CGFLG-C_6_-[KTVRTSADE] **13** were performed in PC3 cell line after 2 h of incubation using positive control (doxorubicin) and negative control (5% DMSO). Different concentrations (0.01, 0.1, 1, 10, and 100 µM) of Dox, peptide **9**, and conjugate **13** were prepared in 5% DMSO and used in the screening in the PC3 cell line after incubation for 2 h to observe the cytotoxicity of targeting peptide **9** in comparison to parent drug Dox and its conjugate **13** (Figure 8). The conjugate **13**, peptide **9**, and Dox showed no significant toxicity at or below 10 µM, possibly due to the shorter incubation time of 2 h. Dox usually requires 24 to 72 h incubation time to show any cytotoxicity at a lower concentration. The targeting peptide **9** did not show any toxicity up to the concentration of 100 µM. 

**Figure 9:**
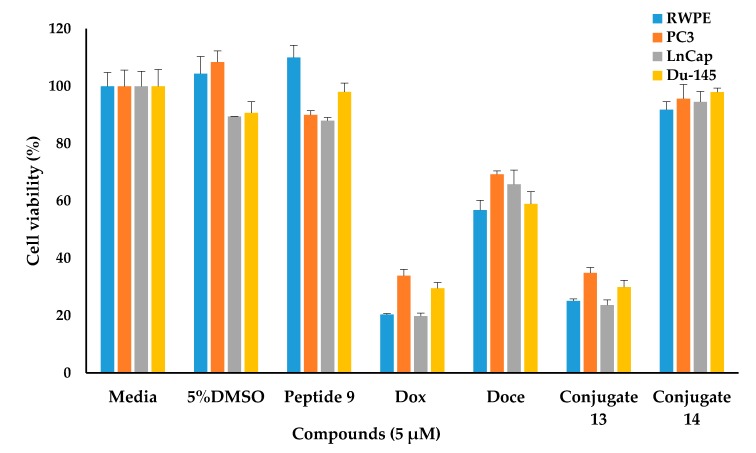
Cell viability of peptide **9**, Dox, Doce, Dox–peptide conjugate **13**, and Doce–peptide conjugate **14** at 5 μM on prostate epithelial and PCa cell lines (RWPE-1, PC3, LNCap, Du-145) after 72 h using MTS assay.

The conjugates **13** and **14** were tested at 5 µM concentration using both positive and negative controls in all the four cell lines after 72 h incubation. Figure 9 depicted that Dox conjugate **13** was moderately toxic with a reduced cell proliferation to a range of 25–35% as compared to Dox which reduced cell proliferation in the range of 20–34% for all selected four cell lines. However, it was interesting to observe that Doce conjugate **14** was almost nontoxic (cell proliferation within the range of 89–96%) in all the cell lines as compared to Doce alone which reduced the cell proliferation in the range of 54–61% (Figure 9). The comparable potency of Dox conjugate **13** versus Dox suggests the possible reduction of disulfide linkage, and release of an active form of Dox from the conjugate after the cellular uptake. As described above in the stability studies, compound **13** was found to be unstable under redox conditions, and in the presence of cathepsin B. On the other hand, the inactivity of conjugate **14** is possibly due to lack of hydrolysis to get an active drug, Doce. Conjugate **14** does not have disulfide linkage and it is possible that ester bond of the secondary alcohol and amide bond do not undergo fast hydrolysis. 

**Figure 10:**
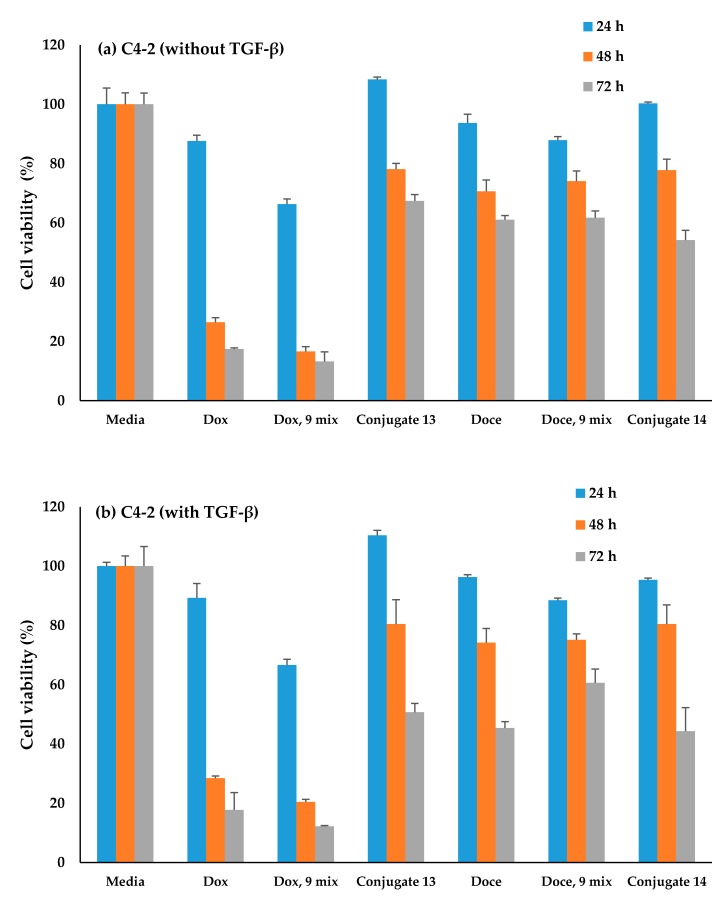
Time-dependent (24, 48, 72 h) cell viability studies of drugs, peptide 9, and drug–peptide conjugates **13**, **14**, and physical mixtures of drug and peptides using MTS on (**a**) prostate C4-2 cell line, a subline of LNCaP without TGF-β, (**b**) with TGF-β treatment.

Furthermore, to evaluate the effect of selectivity of synthesized conjugates containing targeting ligand, the cells were treated with TGF-β for 3 days to induce overexpression of EDB-FN as reported and assayed using cell viability assay for 24–72 h [32]. Also, an equimolar amount of the physical mixture of drug and peptides were evaluated to determine the potency of these compounds in TGF-β positive treated cells. C4-2 cell line was used with and without TGF-β treatment (Figure 10a,b) as it was reported to show overexpression of EDB-FN [31]. C4-2 cells were derived from human prostate adenocarcinoma LNCaP cells. Figure 10a showed an increase in the cytotoxicity of Dox and Dox/peptide **9** physical mixture as compared to the conjugate **13** over an incubation period of 24 h to 72 h. Conjugates **13** and **14** were found to be less cytotoxic as compared to drug alone in 24–72 h. These cells were not treated with TGF-β, so very minimal or no overexpression of EDB-FN. Figure 10b showed the effect of overexpression of EDB-FN in the cell viability. There was no observed effect of TGF-β treatment for the cytotoxicity of Dox and physical mixture of Dox/peptide **9** on the cell viability as compared to the TGF-β untreated cell lines. However, conjugate **13** showed a decrease in cell viability by 17% after 72 h as compared to untreated cell lines. Similarly, Doce and Doce conjugate **14** showed decrease in cell viability by 16 and 10%, respectively, after 72 h. The physical mixtures of Doce/peptide **9** showed a decrease in cell viability by 16% as compared to untreated cells. 

#### 2.2.2. Confocal Microscopy of the Fluorescently Tagged Compounds **17** and **18**

**Figure 11:**
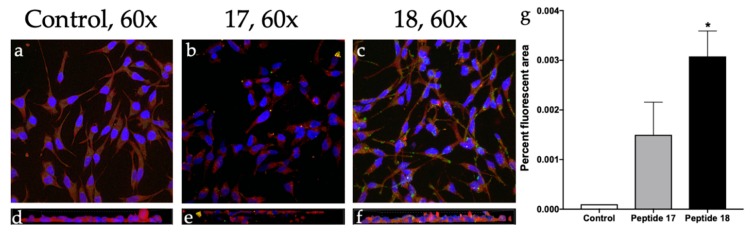
Comparative confocal microscopy of Fluorescent tagged peptide **17** and **18**. Control (**a**) shows no treatment; (**b**) shows FAM-linked disulfide cyclized targeting moiety in the peptide **17**; (**c**) shows FAM linked amide cyclized targeting moiety in the peptide **18**; (**d**), (**e**), and (**f**) are Z-slices of images in (**a**), (**b**), and (**c**); (**g**) shows the unpaired *t*-statistic reflects peptide **18** has higher binding to EDB-FN as compared to peptide **17**.

To evaluate the comparative EDB-FN binding capability of disulfide cyclized peptide **1** and its more stable analog **8**, we prepared the corresponding FAM-conjugated fluorescent tagged derivatives, namely peptide **17** and **18**, respectively. C4-2 cells were used for this assay due to overexpression of EDB-FN [31]. Expression of EDB-FN was confirmed using PCR. The C4-2 cells were incubated with the FAM-conjugated peptide **17** and **18** dissolved in the cell culture media for 12 h. Figure 11 shows the confocal images of EDB-FN stained cells without any peptide (a, control), incubated with peptide **17** (b) and peptide **18** (c) respectively at 60× magnification. As it is evident from Figure 11, peptide **18** showed notably higher binding and colocalization for EDB-FN as compared to peptide **17**. A binary contrast quantification showed a significant increase of 48% in the peptide-stained area in cells incubated with peptide **18** compared to that of peptide **17**. These images reflect binding of peptide **17** and **18** to EDB-FN, which includes linkers and spacer, which presumably did not change the affinity of ligands for EDB-FN. The enhanced binding of peptide **18** over peptide **17** was speculated to be due to the higher stability of ligand 18.

## 3. Materials and Methods

### 3.1. Chemistry 

All amino acids and resins were purchased from AAPPTec LLC (Louisville, KY, USA), Chem-Impex International Inc. (Wood Dale, IL, USA), and CEM Corporation (Matthews, NC, USA). All organic solvents were purchased from Millipore Sigma Corporation (St. Louis, MO, USA), Fischer Scientific (Pittsburgh, PA, USA), and Gyros Protein Technologies, Inc (Tucson, AZ, USA). Anticancer agents Doxorubicin (Dox) and Docetaxel (Doce) were purchased from LC laboratories (Woburn, MA, USA). Masses of intermediate and final products were confirmed by high-resolution matrix-assisted laser desorption/ ionization time-of-flight (MALDI-TOF) mass spectrometer from Bruker Inc. (GT 0264, Billerica, MA, USA) or Applied Biosystems (4800 MALDI TOF/TOF Analyzer, Foster City, CA, USA). Intermediate and final compounds were purified by reversed-phase high-performance liquid chromatography (RP-HPLC) from Shimadzu (Prominence, Columbia, MD, USA) using a gradient system of acetonitrile and water with 0.1% trifluoroacetic acid using reverse phase C18 column (XBridge BEH130 Prep C18), from Waters Corporation (Milford, MA, USA). 5(6)-Carboxyfluorescein diisobutyrate (CFDI) was used to synthesize fluorescently-label peptide (USBiological Life Science, Swampscott, MA, USA). 

#### 3.1.1. Synthesis of Peptide 1 ([CTVRTSADC])

The linear peptide containing nine amino acids (CTVRTSADC) was synthesized by Fmoc/tBu solid-phase peptide synthesis. Rink Amide resin (0.3 mmol, 526 mg, 0.57 mmol/g) was swollen in DMF. The Fmoc group was deprotected using 20% piperidine/DMF under nitrogen for two times (20 min × 2). Side chain-protected amino acids (0.9 mmol) were coupled using HBTU (341 mg, 0.9 mmol, 3 equiv) and DIPEA (313 μL, 1.8 mmol) in DMF as coupling and activating reagent for 90 min. The Fmoc group was deprotected using 20% piperidine/DMF under nitrogen twice (20 min × 2). The peptide was assembled on the resin following coupling of amino acid in the order, e.g., Fmoc-Cys(Trt)-OH (527 mg, 0.9 mmol), Fmoc-Asp(OtBu)-OH (370 mg, 0.9 mmol), Fmoc-Ala-OH (280 mg, 0.9 mmol), Fmoc-Ser(tBu)-OH (345 mg, 0.9 mmol), Fmoc-Thr(Trt)-OH (357 mg, 0.9 mmol), Fmoc-Arg(Pbf)-OH (583 mg, 0.9 mmol), Fmoc-Val-OH (305 mg, 0.9 mmol), Fmoc-Thr(Trt)-OH (357 mg, 0.9 mmol), and Fmoc-Cys(Trt)-OH (527 mg, 0.9 mmol). Coupling and deprotection cycles were repeated to assemble the sequence of the linear protected peptide. Then, the side-chain deprotection and cleavage from the resin were carried out by a freshly prepared cleavage cocktail, trifluoroacetic acid:triisopropylsilane:water (TFA:TIS:H_2_O; 95:2.5:2.5, *v/v/v*, 13.5 mL:750 µL:750 µL,15 mL) for 2 h. The crude peptide was precipitated by the addition of cold diethyl ether (30 mL × 3 times, Et_2_O) and purified by RP- HPLC on a water XBridge BEH130 Prep C18 OBD 10 μm ODS reversed-phase column (2.1 cm × 25 cm) using a gradient system. The crude peptide was purified at a flow rate of 10.0 mL/min using a gradient of 0–100% acetonitrile (0.1% TFA) and water (0.1% TFA) over 60 min on RP-HPLC and then was lyophilized to obtain the linear peptide. Linear peptide (CTVRTSADC): MALDI-TOF (*m*/*z*): C_35_H_64_N_13_O_14_S_2_, calcd. [M + H]^+^ 954.4137; found 954.5512. Cyclization of the linear peptide was carried out by exposing the peptide in 10% DMSO to air at pH 7.4, followed by purification using RP-HPLC. Peptide **1** ([CTVRTSADC]); MALDI-TOF (*m*/*z*): C_35_H_62_N_13_O_14_S_2_, calcd. [M + H]^+^ 952.3980; found 952.4786.

#### 3.1.2. Synthesis of Peptide 8 ([KTVRTSADE])

The linear peptide containing sequence (KTVRTSADE) was synthesized by Fmoc/tBu solid-phase peptide synthesis. Rink amide resin (0.3 mmol, 526 mg, 0.57 mmol/g) was swelled in DMF. The Fmoc group was deprotected using 20% piperidine/DMF under nitrogen for two times (20 min × 2). Amino acids were coupled using HBTU (341 mg, 0.9 mmol, 3 equiv) and DIPEA (313 μL, 1.8 mmol) in DMF as coupling and activating reagents, respectively. The peptide was assembled on the resin following coupling of amino acid in the order, e.g., Fmoc-Glu(Phipr)-OH (438 mg, 0.9 mmol), Fmoc-Asp(OtBu)-OH (370 mg, 0.9 mmol), Fmoc-Ala-OH (280 mg, 0.9 mmol), Fmoc-Ser(tBu)-OH (345 mg, 0.9 mmol), Fmoc-Thr(Trt)-OH (357 mg, 0.9 mmol), Fmoc-Arg(Pbf)-OH (583 mg, 0.9 mmol), Fmoc-Val-OH (305 mg, 0.9 mmol), Fmoc-Thr(Trt)-OH (357 mg, 0.9 mmol), and Fmoc-Lys(Dde)-OH (479 mg, 0.9 mmol). Coupling and deprotection cycles were repeated to assemble the sequence of the linear protected peptide. Then, selective side-chain deprotection were performed in order to remove Phipr (1% TFA/DMF, *v/v*, 15 min × 3 times) on glutamic acid and then Dde (2% hydrazine monohydrate/DMF, 20 min × 2 times) to achieve carboxylic group on the side chain of glutamic acid and amine group at the side chain of lysine side chain amine group. Cyclization of carboxylic acid and the amino group in the side chain was performed using 1-[bis(dimethylamino)methylene]-1H-1,2,3-triazolo[4,5-b]pyridinium 3-oxid hexafluorophosphate (HATU; 344 mg, 0.9 mmol), ethyl cyano(hydroxyimino)acetate (Oxyma Pure; 136 mg, 0.96 mmol), and DIPEA (313 μL, 1.8 mmol) in DMF for 3 h. After cyclization was confirmed by MALDI mass, the cleavage of peptide from the resin was carried out by agitating to a freshly prepared cleavage cocktail, TFA:TIS:H_2_O (95:2.5:2.5, *v/v/v*, 13.5 mL:750 µL:750 µL,15 mL) for 2 h. The crude peptide was precipitated by the addition of cold diethyl ether (30 mL × 3 times, Et_2_O). Peptide **8** ([KTVRTSADE]): MALDI-TOF (*m*/*z*): C_40_H_71_N_14_O_15_, calcd. [M + H]^+^ 987.5223; found 987.2699.

#### 3.1.3. Synthesis of Peptide 5 (Hydrazine-Glutarate-GG-FK-C_6_-[CTVRTSADC])

Peptide **1** on solid support from step 3.1.1 was used before final cleavage to attach a spacer Fmoc-6-Ahx-OH (C_6_) (157 mg, 0.9 mmol, 3 equiv) in the presence of HBTU (341 mg, 0.9 mmol, 3 equiv) and DIPEA (313 μL, 1.8 mmol, 6 equiv) in DMF as coupling and activating reagents for 3 days. The resin was washed, and Fmoc-Lys(Boc)-OH (421 mg, 0.9 mmol) and Fmoc-Phe-OH (348 mg, 0.9 mmol) were conjugated similarly. Fmoc-Gly-OH (267 mg, 0.9 mmol) was coupled twice and then reacted with glutaric anhydride (102 mg, 0.9 mmol) to provide a spacer and a carboxylic acid functional group for further functionalization, respectively. After washing the resin, Boc-hydrazine (396 mg, 3 mmol), HOBt (405 mg, 3 mmol), EDC (465 mg, 3 mmol), and DIPEA (1.045 mL, 6 mmol) in dry DMF coupled for 48 h to provide hydrazine functional group at the *N* terminal. The resin was washed and cleaved using cleavage cocktail, TFA:TIS:H_2_O (95:2.5:2.5, *v/v/v*, 13.5 mL:750 µL:750 µL,15 mL) for 2 h to afford linear peptide **5**, which after RP-HPLC purification and cyclization in 10% DMSO under air afforded cyclic peptide **5**. MALDI-TOF (*m*/*z*): C_65_H_111_N_21_O_21_S_2_ calcd. [M + H]^+^ 1585.7704; found 1585.5270.

#### 3.1.4. Synthesis of Peptide 6 (Hydrazine-Glutarate-GG-VCit-C_6_-[CTVRTSADC])

The synthesis of peptide **6** was followed by using a similar procedure described in 3.1.3 upto the coupling of Fmoc-6-Ahx-OH, and then Fmoc-L-citrulline-OH (Cit; 476 mg, 1.2 mmol) and Fmoc-Val-OH (305 mg, 0.9 mmol) were coupled on the solid phase. After washing and deprotecting Fmoc group, the resin was further reacted with Fmoc-Gly-OH (twice), glutaric anhydride, and then Boc-hydrazine as mentioned in the 3.1.3. The cleavage of linear peptide with cleavage cocktail, TFA:TIS:H_2_O (95:2.5:2.5, *v/v/v*, 13.5mL:750µL:750µL, 15 mL), purification by RP-HPLC, and cyclization under 10% DMSO under air afforded peptide **6**. MALDI-TOF (*m*/*z*): C_61_H_109_N_22_O_22_S_2_, calcd. [M + H]^+^ 1565.7527; found 1565.5920. 

#### 3.1.5. Synthesis of Peptides 19 (C-GFLG-C_6_-[CTVRTSADC]) and 9 (C-GFLG-C_6_-[KTVRTSADE]) 

Both the peptidyl resin from synthesis of peptide **1** (from step 3.1.1) and peptide **8** (from step 3.1.2) were further reacted to attach a Fmoc-6-Ahx-OH (157 mg, 0.9 mmol), Fmoc-Gly-OH (267 mg, 0.9 mmol), Fmoc-Leu-OH (318 mg, 0.9 mmol), Fmoc-Phe-OH (348 mg, 0.9 mmol), Fmoc-Gly-OH (267 mg, 0.9 mmol), and Fmoc-Cys(Trt)-OH (527 mg, 0.9 mmol) amino acid residues in the separate peptide synthesis vessel. Coupling and deprotection cycles were repeated to assemble the sequence of the linear protected peptide followed by cleavage with cleavage cocktail, TFA:TIS:H_2_O (95:2.5:2.5, *v/v/v*, 13.5 mL:750 µL:750 µL,15 mL) for 2 h to afford complete form of peptide **9** and linear form of peptide **19** which further cyclized using 10% DMSO/H_2_O. Peptides were purified using RP-HPLC and characterized using MALDI TOF. Peptide **19** (C-GFLG-C_6_-[CTVRTSADC]); MALDI-TOF (*m*/*z*): C_63_H_106_N_19_O_20_S_2_, calcd. [M + H]^+^ 1544.6863; found 1544.1794; Peptide **9** (C-GFLG-C_6_-[KTVRTSADE]); MALDI-TOF (*m*/*z*): C_68_H_113_N_20_O_21_S, calcd. [M]^+^ 1577.8110; found 1577.6815.

#### 3.1.6. Synthesis of Conjugate 12 (Dox-hydrazone-glutarate-GG-VCit-C_6_-[CTVRTSADC]) 

A solution of dox (16.3 mg, 0.03 mmol) was prepared after dissolving in anhydrous methanol (2 mL) and was added dropwise to the solution of peptide **5** (47.3 mg, 0.03 mmol) in anhydrous methanol (1 mL) followed by addition of TFA (0.1%, 3 µL) to acidify the reaction mixture with continued stirring overnight at room temperature in 5 mL amber glass vial. The solvent was removed under reduced pressure and conjugate was purified by RP-HPLC. Conjugate **12** (Dox-hydrazone-glutarate-GG-VCit-C_6_-[CTVRTSADC]); MALDI (*m*/*z*): C_88_H_136_N_23_O_32_S_2_, calcd. [M + H]^+^ 2090.9003; found: 2090.7130.

#### 3.1.7. Synthesis of Conjugate 13 (Dox-s-s-CGFLG-C_6_-[KTVRTSADE]) 

First, dox thiol (Dox-SH) was prepared by reacting Dox (25 mg, 43 μmol) with 2-iminothiolane (2.5 equiv, 107 μmol) in methanol (25 mL) and triethylamine (TEA) (5 equiv, 30 µL) stirred at room temperature (7 h). The reaction mixture was precipitated by ether (15 mL), and upon decantation, the precipitant was evaporated using rotary evaporator. The sulfhydryl group of Dox was activated with dithiodipyridine by reacting Dox-SH (1 equiv, 27 mg, 43 µmol) with 2,2′-dithiodipyridine (2.5 equiv, 50 mg, 107 µmol) in methanol (0.1% acetic acid, *v/v*) with stirring at room temperature for overnight. The resulting precipitate was washed again with cold ether and dried to afford compound (Dox-S-S-Pyridine). A single peak was detected in the analytical HPLC. Activated Dox compound **10** was coupled with peptide **9** (47 mg, 0.03 mmol) via disulfide bridge using overnight stirring in the degassed water (5 mL). The solvent was evaporated, and peptide **13** was purified using RP-HPLC. Conjugate **13** (Dox-s-s-CGFLG-C_6_-[KTVRTSADE]); MALDI-TOF (m/z): C_99_H_150_N_22_O_32_S_2_, calcd. [M + H]^+^ 2223.0227; found 2222.3398.

#### 3.1.8. Synthesis of Conjugate 14 (Doce-βA-thioether-CGFLG-C_6_-[KTVRTSADE])

Doce-βA-NH_2_ was first prepared with the stirring of Docetaxel (5 mg, 0.012 mmol), Fmoc-β-alanine (5.5 mg, 0.062 mmol), using 2-(6-chloro-1-H-benzotriazole-1-yl)-1,1,3,3-tetramethylaminium hexafluorophosphate (HCTU) (14.9 mg, 36 µmol, 3 equiv) and DIPEA (12 µL, 72 µmol, 6 equiv) in anhydrous DMF (1.5 mL), at room temperature for 90 min. Fmoc was removed by treating with 40% piperidine in DMF (*v/v*, 10 mL, 20 min), and the resulting peptide was precipitated by ether. Purification was performed using RP-HPLC followed by lyophilization. Doce-βA-NH_2_ (10.8 mg, 10 µmol) was reacted with sulfosuccinimidyl-4-(N-maleimidomethyl)cyclohexane-1-carboxylate (sulfo-SMCC) (6.5 mg, 15 µmol, 1.5 equiv) in *N*,*N’*-diisopropylcarbodiimide (DIC, 8 µL, 5 equiv) and TEA (8 µL, 6 equiv) using methanol (1 mL), overnight. Docetaxel-βA-SMCC (**11**) was purified using RP-HPLC. The Docetaxel-βA-SMCC (7.6 mg, 7 µmol) was dissolved in DMF (5 mL), reacted with peptide **9** (C-GFLG-C_6_-[KTVRTSADE]) (12 mg, 76 µmol, 1.08 equiv) in PBS with 20 mM EDTA at pH 8 (20 mL) for 17 h at room temperature. The formation of conjugated compound **14** (Doce-*β*A-thioether-C-GFLG-C_6_-[KTVRTSADE]) was confirmed by mass analysis using MALDI; MALDI-TOF (*m*/*z*): C_126_H_184_N_23_O_39_S, calcd. [M + H]^+^ 2675.2842; found 2675.8490.

#### 3.1.9. Synthesis of FAM Conjugate Peptide 17 (FAM-GFLG-C_6_-[CTVRTSADC])

First, the linear protected peptide **15** (NH_2_-GFLG-C_6_-C(Trt)T(tBu)VR(Pbf)T(tBu)S(tBu)AD(OtBu)C(Trt)) on Rink amide resin was synthesized as described above at 0.2 mmol (350 mg) scale using Rink amide resin. The peptidyl-resin was washed in DMF (15 mL, 3 × 2 min). The coupling of 5(6)-carboxyfluorescein diisobutyrate (CFDI, 2.5 equiv, 258 mg), in the presence of HOAt (2.5 equiv, 68 mg), (7-azabenzotriazol-1-yloxy)tripyrrolidinophosphonium hexafluorophosphate (PyAOP, 2.5 equiv, 260 mg), and DIPEA (5 equiv, 174 μL) in anhydrous DMF (12 mL) was carried out for 4.5 h followed by washing with DMF. The deprotection of isobutyrate protection in CFDI was carried out by agitating the peptidyl resin using piperidine (20%, *v/v*, 25 mL, 2 × 15 min). The resin was finally washed with DMF (3 × 3 mL), DCM (3 × 3 mL), and MeOH (3 × 3 mL) and finally dried in vacuum for 30 min. The fluorescent-labeled peptide was cleaved using TFA:TIS:H_2_O (95:2.5:2.5, *v/v/v,* 6.75 mL:375 μL:375 μL) for 2 h. The crude peptide was precipitated with cold diethyl ether (10 mL) and centrifuged (4500 rpm, 6 min). The solvent was decanted to obtain the precipitant, dried under nitrogen which was further dissolved in 10% DMSO (100 mL) under dark to form disulfide bridge between cysteine’s thiol residues under air for overnight. The solvent was evaporated under reduced pressure to afford viscous peptide precipitate. The precipitate was dissolved in 30% ACN/H_2_O (*v/v*, 4 mL), purified by using RP-HPLC, and lyophilized. The mass was confirmed for peptide **17** with MALDI-TOF (*m*/*z*); C_81_H_1_N_18_O_25_S_2_, calcd. [M + H]^+^ 1799.7249; found 1799.9280.

#### 3.1.10. Synthesis of FAM Conjugate Peptide 18 (FAM-GFLG-C_6_-[KTVRTSADE])

The cyclized protected peptide [KT(tBu)VR(Pbf)T(tBu)S(tBu)AD(OtBu)E] was assembled on Rink amide resin (0.2 mmol, 350 mg) using steps in Section 3.1.2. The peptidyl resin was washed in DMF (15 mL, 3 × 2 min) and then solvents were filtered off. Then, Fmoc-Ahx-OH, Fmoc-Gly-OH, Fmoc-Leu-OH, Fmoc-Phe-OH, and Fmoc-Gly-OH were coupled as mentioned above, followed by resin washing with DMF (15 mL, 3 × 2 min). The deprotection of *N*-terminal Fmoc was carried out using 20% piperidine in DMF (*v/v*, 2 × 10 min) and then washed with DMF (9 mL, 3 × 2 min) to afford intermediate peptide **16** (NH_2_-C(Trt)GFLG-C_6_-[KT(tBu)VR(Pbf)T(tBu)S(tBu)AD(OtBu)E]-Rink amide resin). The coupling of 5(6)-carboxyfluorescein diisobutyrate (CFDI, 2.5 equiv, 258 mg), in the presence of HOAt (2.5 equiv, 68 mg), (7-azabenzotriazol-1-yloxy)tripyrrolidinophosphonium hexafluorophosphate (PyAOP, 2.5 equiv, 260 mg), and DIPEA (5 equiv, 174 μL) in anhydrous DMF (12 mL) was carried out to the peptidyl resin for 4.5 h followed by washing with DMF. The deprotection of isobutyrate protection in CFDI was carried out by agitating the peptidyl resin using 20% piperidine/DMF (*v/v*, 25 mL, 2 × 15 min). The resin was finally washed with DMF (3 × 3 mL), DCM (3 × 3 mL), and MeOH (3 × 3 mL) and finally dried under vacuum for 30 min. The fluorescent-labeled peptide was cleaved using TFA:TIS:H_2_O (95:2.5:2.5, *v/v/v,* 6.75 mL:375 μL:375 μL) for 2 h. The crude peptide was precipitated with cold diethyl ether (10 mL), centrifuged (4500 rpm, 6 min). The solvent was decanted to obtain the precipitant to afford viscous peptide precipitate. The precipitate was dissolved in ~30% ACN/H_2_O (*v/v*, 4 mL), purified by using RP-HPLC, and lyophilized. The mass was confirmed for peptide **18** with MALDI-TOF (m/z); C_86_H_118_N_19_O_26_, calcd. [M + H]^+^ 1832.8495; found 1832.3740.

### 3.2. Stability in PBS and Dithiothreitol (DTT)

The stability of peptide–drug conjugate **13** was evaluated in the phosphate buffered saline (PBS). The pH of PBS was adjusted to 6.8, 7.0, 7.4 using HCl and NaOH as needed. In brief, 250 µL of 1 × PBS was mixed with 45 µL of 0.25 mM Dox-peptide conjugate **13**. The mixture was incubated at 37 °C in a water bath covered with aluminum foil. An aliquot (40 µL) was removed at different time intervals, such as 4, 24, and 84 h and analyzed using analytical RP-HPLC at 490 nm. The percentage of the remained peptide was calculated using the area under the curve (AUC) by integrating the chromatogram in analytical HPLC. For evaluation of stability in DTT, a solution of DTT (0.120 mM) was prepared using PBS (pH 7.4). A solution of compounds (**1, 8**, and **13** at 1 mM) was prepared in PBS (pH = 7.4). A volume of 45 µL of the peptide was added to 2.250 mL of PBS in a UV-blocking glass vial. The glass vials were put under nitrogen and stirred using a small magnetic bead. A volume of DTT (222 mg, 12 equiv) was added to the solution, which was incubated with each peptide sample to obtain a working concentration of 0.12 mM DTT and 0.010 mM peptide. Aliquots of 200 µL were collected at different time intervals and analyzed using analytical HPLC vials (0 min was used as controls without DTT) after quenching the DTT with 10 µL of H_2_O (pH = 2). The aliquots were run on analytical HPLC with detection at 214 nm. 

### 3.3. Stability in Human Serum 

The stability was performed using 25% human serum. 325 µL of RPMI-1640 media was incubated with 125 µL of human serum at 37 °C in a water bath. Then 50 µL of 125 µM of conjugate **13** was added to the RPMI and serum. The control experiment was performed without conjugate using an aqueous 5% DMSO. Several aliquots (55 µL) were removed at 2, 5, 10, 15, 30, 60, 90, and 120 min. The aliquots were added to 120 µL of cold methanol to precipitate the serum proteins with vigorous mixing using vortex and kept at ice for 10 min. Then, the samples were centrifuged at 17× *g* (13,300 rpm) for 10 min to get a pellet of the insoluble peptide. The supernatant was collected and analyzed on analytical HPLC, as mentioned before. 

### 3.4. Hydrolysis Studies with Cathepsin Enzyme

Human cathepsin B (CTSB, Lot # CB2016-02 from Athens Research, Athens, GA, USA) was used to hydrolyze the cathepsin linker GFLG in conjugated peptide **13**. A stock solution of cathepsin B enzyme (0.434 µg/µL) was prepared using 50 mM acetate (pH 5.0) buffer with 1 mM EDTA. The activation buffer was separately prepared using 30 mM DTT and 15 mM EDTA in H_2_O. An aliquot of 2.25 µL of cathepsin B stock was activated with 5 µL of activation buffer for 15 min at room temperature then diluted with 1.185 mL of acetate buffer and aliquoted into different tubes with 200 µL each. Further 8 µL of 1 mM of conjugated peptide **13** was added to aliquots and incubated for various time intervals. Then, the reaction was quenched by boiling to denature cathepsin B for 10 min. A 400 µL of cold methanol was added to precipitate the proteins followed by centrifugation of sample 17× *g* (13,300 rpm) for 10 min. A supernatant (500 µL) was collected, evaporated, and reconstituted with 300 µL of HPLC solvent A (H_2_O with 0.1% TFA). The eluent fraction from HPLC was further collected and analyzed using MALDI-TOF mass spectrometry to confirm the hydrolysis of the conjugate.

### 3.5. Cell Culture 

Human epithelial prostate cell line (RWPE-1, ATCC No. CRL-11610), human prostate cancer cell line (Human prostate carcinoma DU-145, ATCC No. HTB-81), human adenocarcinoma PC3, ATCC No. CRL-1435), androgen-sensitive human prostate adenocarcinoma cells (LNCaP, ATCC No. CRL-1740), and castration-resistant LNCaP-derived C4-2 (ATCC No. CRL-1595) were purchased from American Type Culture Collection (ATCC), Manassas, VA, USA. All the media, serum, and antibiotics were bought from American Type Culture Collection (ATCC) (Manassas, VA, USA) and Sigma life-science (St. Louis, MO, USA). Keratinocytes SFM (1×) with EGF + BPE was bought from Gibco life technologies (Thermo Fisher Scientific), Gaithersburg, MD, USA. CellTiter 96 was bought from Promega, Madison, WI, USA. Dithiothreitol (DTT) was bought from Thermo Fisher scientific, Carlsbad, CA, USA, and TGF-β was bought from Abcam, Boston, MA, USA.

#### 3.5.1. Cell Viability Assays using MTS

Cell-proliferation assay using MTS reagent was conducted against five cell lines (RWPE-1, PC3, DU-145, LNCaP, and, C4-2). Cells were seeded into 96-well plates (5 × 10^3^ cells for all the cells) and incubated with 100 µL of complete medium (RPMI-1640 for RWPE-1, DU-145, LNCaP, and C4-2; DMEM for PC3) overnight at 37 °C with 5% CO_2_. Various concentrations (0, 0.06, 0.6, 6, 60, 600 μM) of the peptide solution (20 μL) were added to cells to yield the final concentrations of peptide (0, 0.01, 0.1, 1, 10, 100 μM). The cells were kept in an incubator (37 °C, 5% CO_2_) for 24, 48, or 72 h accordingly for different treatment time responses. Then, a CellTiter 96 aqueous solution (20 μL) was added to each well and incubated for 4 h under the same condition. The absorbance was obtained at 490 nm using SpectraMax M2 microplate reader to detect the formazan product. Wells containing cells in the absence of any peptide were used as a control. The percentage of cell viability was calculated by [(OD value of cells treated with the test mixture of compounds) − (OD value of culture medium)] / [(OD value of control cells) − (OD value of culture medium)] × 100%. For other time-dependent experiments, compounds (5 μM) were added to each well in triplicate and kept for 24, 48, and 72 h at 37 °C in a 5% CO_2_ incubator. Cell viability was determined by measuring the absorbance at 490 nm using SpectraMax M2 microplate spectrophotometer, as mentioned above.

#### 3.5.2. Overexpression of EDB-FN using TGF-β

To compare the cytotoxicity of compounds using treatment of TGF-β, the cells were culture, and 8000 cells (RPWE-1, PC3, DU-145, LNCap, and C4-2) were added in 120 μL per well with TGF-β treatment. Cells were exposed to TGF-β at 10 ng/mL for 3 days. Then cells were seeded in the corresponding medium (DMEM containing 10% FBS and 1% penicillin–streptomycin for DU-145, LNCaP, and PC3; keratinocyte containing 0.1% FBS without antibiotics for RWPE-1), 24 h prior to the experiment. Then, MTS assay was followed up, as mentioned in 3.5.1. 

#### 3.5.3. Confocal Microscopy 

Prostate cancer cell line (C4-2) was grown in RPMI medium containing 10% fetal bovine serum and 1% penicillin/streptomycin at the 37 °C in a 5% CO_2_ incubator. The culture medium was changed every 2–3 days until confluency was reached to 80%. Each cells group was treated with 5 μg of fluorescence-tagged peptides (17 and 18) for 12 h. A set of control groups was also prepared, which was not treated with peptides. Then all cells were fixed with 4% paraformaldehyde for 10 min and washed for 10 min (3 times with PBS). The cells were blocked in 1% FBS/PBS for 10 min and washed for 10 min (3 times with PBS). To target EDB-FN on the cell surface, the cells were incubated with a mouse monoclonal anti-fibronectin antibody (Abcam, Cambridge, MA, USA) (1:1000 dilution using 1% FBS/PBS) overnight at the 4 °C. The cells were washed for 10 min (3 times with PBS), and a Texas red-conjugated anti-mouse secondary antibody was used in 1% FBS/PBS (5:1000 dilution) at the room temperature for 1 h. Then cells were washed for 10 min (3 times with PBS), and at the end, nuclei were stained using DAPI and covered with coverslips. Representative images of cells were captured using a standard confocal microscope (Nikon Eclipse Ti-E, Nikon Instruments Inc., Melville, NY, USA) at 60× magnification, and the images were processed for binary contrast quantification. 

## 4. Conclusions

In conclusion, the synthesis and optimization of EDB-FN ligands were achieved using Fmoc/tBu solid phase synthesis. The lactam cyclized ligand [KTVRTSADE] **8** showed higher stability under redox condition (8 h) as compared to disulfide cyclized ligand [CTVRTSADC] **1** (2.6 min). A half-life of 11.8 min was detected for Dox conjugate **13** during treatment with 25% serum. A cathepsin enzyme based linkers were introduced in Dox/Doce conjugates (**13** and **14**), which releases the drug from conjugate within 5 min after treatment with cathepsin enzyme. The antiproliferative activity of Dox conjugate **13** exhibited comparable or the similar antiproliferative activity versus Dox alone or physical mixture in 72 h in all the cell lines (RWPE-1, PC3, DU-145, and LNCaP). The Doce conjugate **14** showed mild or no activity which might be due to the lack of hydrolysis of the drug from conjugate in all the cell lines. The TGF-β treatment in C4-2 cell lines demonstrates the effect of overexpression of EDB-FN on cell viability. Confocal microscopy demonstrated localization of ligands at EDB-FN in C4-2 cell line. The peptide **18** containing lactam cyclization showed higher targeting at EDB-FN in C4-2 cell lines as compared to peptide **17** containing disulfide cyclization. These studies provide a better understanding for designing peptide–drug conjugate to be used as a chemotherapeutic agent against PCa.

## Figures and Tables

**Table 1:** The sequences of extra domain B of fibronectin (EDB-FN) targeting peptides and peptide–drug conjugates.

Peptide	Peptide Sequence	Chemical Formula	Exact Mass	Found (M/Z)
1	[CTVRTSADC]	C_35_H_62_N_13_O_14_S_2_	952.3980	952.4786, [M + H]^+^
5	Hydrz^b^-Glutrt^b^-GG-FK-C_6_^a^-[CTVRTSADC]	C_65_H_111_N_21_O_21_S_2_	1585.7704	1585.5270, [M +H]^+^
6	Hydrz^b^-Glutrt^b^-GG–VCita–C_6_^a^-[CTVRTSADC]	C_61_H_109_N_22_O_22_S_2_	1565.7527	1565.5920, [M + H]^+^
8	[KTVRTSADE]	C_40_H_71_N_14_O_15_	987.5223	987.2699, [M + H]^+^
9	CGFLG-C_6_^a^-[KTVRTSADE]	C_68_H_113_N_20_O_21_S	1577.8110	1577.6815, [M]^+^
10	Dox-s-s-Pyridine	C_36_H_40_N_3_O_11_S_2_	754.2104	754.4053, [M + H]^+^
11	Doce-βA-SMCC	C_58_H_72_N_3_O_18_	1098.4811	1098.1675, [M + H]^+^
12	Dox-hydrz^c^-Glutrt^b^–GG–VCit^a^–C_6_^a^-[CTVRTSADC]	C_88_H_136_N_23_O_32_S_2_	2090.9003	2090.7130, [M + H]^+^
13	Dox-s-s-CGFLG-C_6_^a^-[KTVRTSADE]	C_99_H_150_N_22_O_32_S_2_	2223.0227	2222.3398, [M +H]^+^
14	Doce-βA-thioether-CGFLG-C_6_^a^-[KTVRTSADE]	C_126_H_184_N_23_O_39_S	2675.2842	2675.8490, [M + H]^+^
15	GFLG-C_6_^a^-[CTVRTSADC]	C_60_H_100_N_18_O_19_S_2_	1440.6615	1440.5944, [M + H]^+^
16	GFLG-C_6_^a^-[KTVRTSADE]	C_65_H_109_N_19_O_20_	1475.8096	1475.8738, [M + H]^+^
17	FAM-GFLG-C_6_^a^-[CTVRTSADC]	C_81_H_111_N_18_O_25_S_2_	1799.7249	1799.928, [M + H]^+^
18	FAM-GFLG-C_6_^a^-[KTVRTSADE]	C_86_H_118_N_19_O_26_	1832.8495	1832.3740, [M + H]^+^
19	CGFLG-C_6_^a^-[CTVRTSADC]	C_63_H_106_N_19_O_20_S_3_	1544.6863	1544.1794, [M + H]^+^

Cyclic peptides are shown using the [] bracket. ^a^Unnatural amino acids denominated by βA, C_6_, and Cit, which represent beta-alanine, 6-aminohexanoic acid, and citrulline, respectively. FAM stands for 5(6)-carboxyfluorescein. ^b^Hydrazine and glutarate linkers are abbreviated as Hydrz and Glutrt, respectively. ^c^Hydrazone.

## Supplementary Materials

Supplementary materials can be found at https://www.mdpi.com/1422-0067/20/13/3291/s1.

## References

[1] Siegel R.L., Miller K.D., Jemal A. (2018). Cancer statistics, 2018. CA Cancer J. Clin..

[2] American Cancer Society Prostate Cancer. https://www.cancer.org/cancer/prostate-cancer.html.

[3] Gravis G. (2019). Systemic treatment for metastatic prostate cancer. Asian J. Urol..

[4] Evison B.J., Sleebs B.E., Watson K.G., Phillips D.R., Cutts S.M. (2016). Mitoxantrone, More than Just Another Topoisomerase II Poison. Med. Res. Rev..

[5] Summers N., Vanderpuye-Orgle J., Reinhart M., Gallagher M., Sartor O. (2017). Efficacy and safety of post-docetaxel therapies in metastatic castration-resistant prostate cancer: a systematic review of the literature. Curr. Med. Res. Opin..

[6] Alavi M., Hamidi M. (2019). Passive and active targeting in cancer therapy by liposomes and lipid nanoparticles. Drug Metab. Pers. Ther..

[7] Zhang J., Wang L., You X., Xian T., Wu J., Pang J. (2019). Nanoparticle Therapy for Prostate Cancer: Overview and Perspectives. Curr. Top. Med. Chem..

[8] Nasrolahi Shirazi A., Tiwari R., Chhikara B.S., Mandal D., Parang K. (2013). Design and biological evaluation of cell-penetrating peptide–doxorubicin conjugates as prodrugs. Mol. Pharm..

[9] Schally A.V., Nagy A. (1999). Cancer chemotherapy based on targeting of cytotoxic peptide conjugates to their receptors on tumors. Eur. J. Endocrinol..

[10] Arap W., Pasqualini R., Ruoslahti E. (1998). Cancer treatment by targeted drug delivery to tumor vasculature in a mouse model. Science.

[11] Srinivasarao M., Low P.S. (2017). Ligand-targeted drug delivery. Chem. Rev..

[12] Åkerfelt M., Härmä V., Nees M. (2011). Advanced Models for Target Validation & Drug Discovery in Prostate Cancer. Prostate Cancer-From Bench to Bedside.

[13] Bonnans C., Chou J., Werb Z. (2014). Remodelling the extracellular matrix in development and disease. Nat. Rev. Mol. Cell Biol..

[14] Kumra H., Reinhardt D.P. (2016). Fibronectin-targeted drug delivery in cancer. Adv. Drug Deliv. Rev..

[15] Mani S.A., Guo W., Liao M.-J., Eaton E.N., Ayyanan A., Zhou A.Y., Brooks M., Reinhard F., Zhang C.C., Shipitsin M. (2008). The Epithelial-Mesenchymal Transition Generates Cells with Properties of Stem Cells. Cell.

[16] Albrecht M., Renneberg H., Wennemuth G., Möschler O., Janssen M., Aumüller G., Konrad L. (1999). Fibronectin in human prostatic cells in vivo and in vitro: expression, distribution, and pathological significance. Histochem. Cell Biol..

[17] Kaspar M., Zardi L., Neri D. (2006). Fibronectin as target for tumor therapy. Int. J. Cancer.

[18] Han Z., Zhou Z., Shi X., Wang J., Wu X., Sun D., Chen Y., Zhu H., Magi-Galluzzi C., Lu Z.-R. (2015). EDB Fibronectin Specific Peptide for Prostate Cancer Targeting. Bioconjug. Chem..

[19] Staudacher A.H., Brown M.P. (2017). Antibody drug conjugates and bystander killing: is antigen-dependent internalisation required?. Br. J. Cancer.

[20] Dubowchik G.M., Firestone R.A., Padilla L., Willner D., Hofstead S.J., Mosure K., Knipe J.O., Lasch S.J., Trail P.A. (2002). Cathepsin B-Labile Dipeptide Linkers for Lysosomal Release of Doxorubicin from Internalizing Immunoconjugates:  Model Studies of Enzymatic Drug Release and Antigen-Specific In Vitro Anticancer Activity. Bioconjug. Chem..

[21] Shao L.-H., Liu S.-P., Hou J.-X., Zhang Y.-H., Peng C.-W., Zhong Y.-J., Liu X., Liu X.-L., Hong Y.-P., Firestone R.A. (2012). Cathepsin B cleavable novel prodrug Ac-Phe-Lys-PABC-ADM enhances efficacy at reduced toxicity in treating gastric cancer peritoneal carcinomatosis. Cancer.

[22] Zhong Y., Shao L., Li Y. (2013). Cathepsin B-cleavable doxorubicin prodrugs for targeted cancer therapy. Int. J. Oncol..

[23] Tsakalozou E., Eckman A.M., Bae Y. (2012). Combination Effects of Docetaxel and Doxorubicin in Hormone-Refractory Prostate Cancer Cells. Biochem. Res. Int..

[24] Wohl A.R., Michel A.R., Kalscheuer S., Macosko C.W., Panyam J., Hoye T.R. (2014). Silicate esters of paclitaxel and docetaxel: Synthesis, hydrophobicity, hydrolytic stability, cytotoxicity, and prodrug potential. J. Med. Chem..

[25] Skwarczynski M., Hayashi Y., Kiso Y. (2006). Paclitaxel prodrugs: toward smarter delivery of anticancer agents. J. Med. Chem..

[26] Kingston D.G.I. (2001). Taxol, a molecule for all seasons. Chem. Commun..

[27] Inoue T., Cavanaugh P.G., Steck P.A., Brünner N., Nicolson G.L. (1993). Differences in transferrin response and numbers of transferrin receptors in rat and human mammary carcinoma lines of different metastatic potentials. J. Cell. Physiol..

[28] Pan H., Yang J., Kopeckova P., Kopecek J. (2011). Backbone Degradable Multiblock N-(2-Hydroxypropyl)methacrylamide Copolymer Conjugates via Reversible Addition−Fragmentation Chain Transfer Polymerization and Thiol−ene Coupling Reaction. Biomacromolecules.

[29] Cunningham D., You Z. (2015). In vitro and in vivo model systems used in prostate cancer research. J. Biol. Methods.

[30] Tai S., Sun Y., Squires J.M., Zhang H., Oh W.K., Liang C.Z., Huang J. (2011). PC3 is a cell line characteristic of prostatic small cell carcinoma. Prostate.

[31] Bisoffi M., Klima I., Gresko E., Durfee P.N., Hines W.C., Griffith J.K., Studer U.E., Thalmann G.N. (2004). Expression profiles of androgen independent bone metastatic prostate cancer cells indicate up-regulation of the putative serine-threonine kinase GS3955. J. Urol..

[32] Han Z., Lu Z.-R. (2017). Targeting fibronectin for cancer imaging and therapy. J. Mater. Chem. B.

