# Supplementary material for: EDB-FN Targeted Peptide–Drug Conjugates for Use against Prostate Cancer
Source: Int J Mol Sci. 2019 Jul 4;20(13):3291. doi: 10.3390/ijms20133291 (PMC6651341; doi:10.3390/ijms20133291)
Supplement: Supplementary file 1 [file ijms-20-03291-s001.pdf]

Supplementary Information

EDB-FN Targeted Peptide-Drug Conjugates for Use against Prostate Cancer

Shang Eun Park[1], Kiumars Shamloo[1], Timothy A. Kristedja[2], Shaban Darwish[1,3], Marco Bisoffi[1,2], Keykavous Parang[1], Rakesh Kumar Tiwari[1*]

1 Center for Targeted Drug Delivery, Department of Biomedical and Pharmaceutical Sciences, Chapman University School of Pharmacy, Harry and Diane Rinker Health Science Campus, 9401 Jeronimo Road, Irvine, CA 92618, United States; park327@mail.chapman.edu (S.E.P.); shaml103@mail.chapman.edu (KS); krist102@mail.chapman.edu (T.K.); shaban_darwish@yahoo.com.sg (S.D.); bisoffi@chapman.edu (M.B.); parang@chapman.edu (K.P.); tiwari@chapman.edu (R.T.)

2 Biochemistry and Molecular Biology, Schmid College of Science and Technology, Chapman University, Orange, CA 92866, United States

3 Organometallic and Organometalloid Chemistry Department, Chemical Industries Research Division, National Research Centre, 33 EL Bohouth St. (former EL Tahrir st.) Dokki, Giza, Egypt, Postal Code: 12622

* Correspondence: tiwari@chapman.edu; Tel.: +1-714-516-5483

Received: date; Accepted: date; Published: date

Table of Contents: **Page**

1.	Mass spectra of selected synthesized compounds	S2-S19
2.	HPLC spectra of selected compounds	S10, S12, S15
3.	Dox Analytical HPLC Chromatogram	S20
4.	Cytotoxicity of Peptides	S21-S22

1. Mass spectra of synthesized compounds

A: MALDI-TOF of linear peptide (CTVRTSADC)

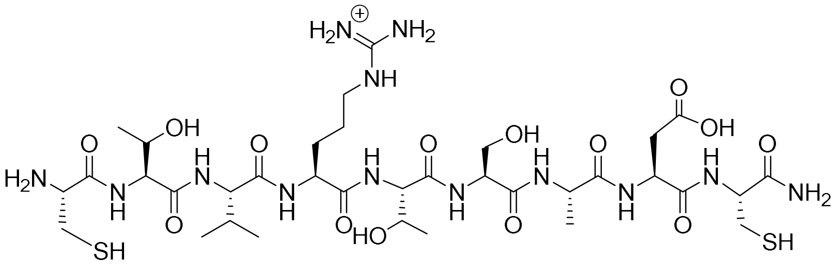

Exact Mass: 954.4132

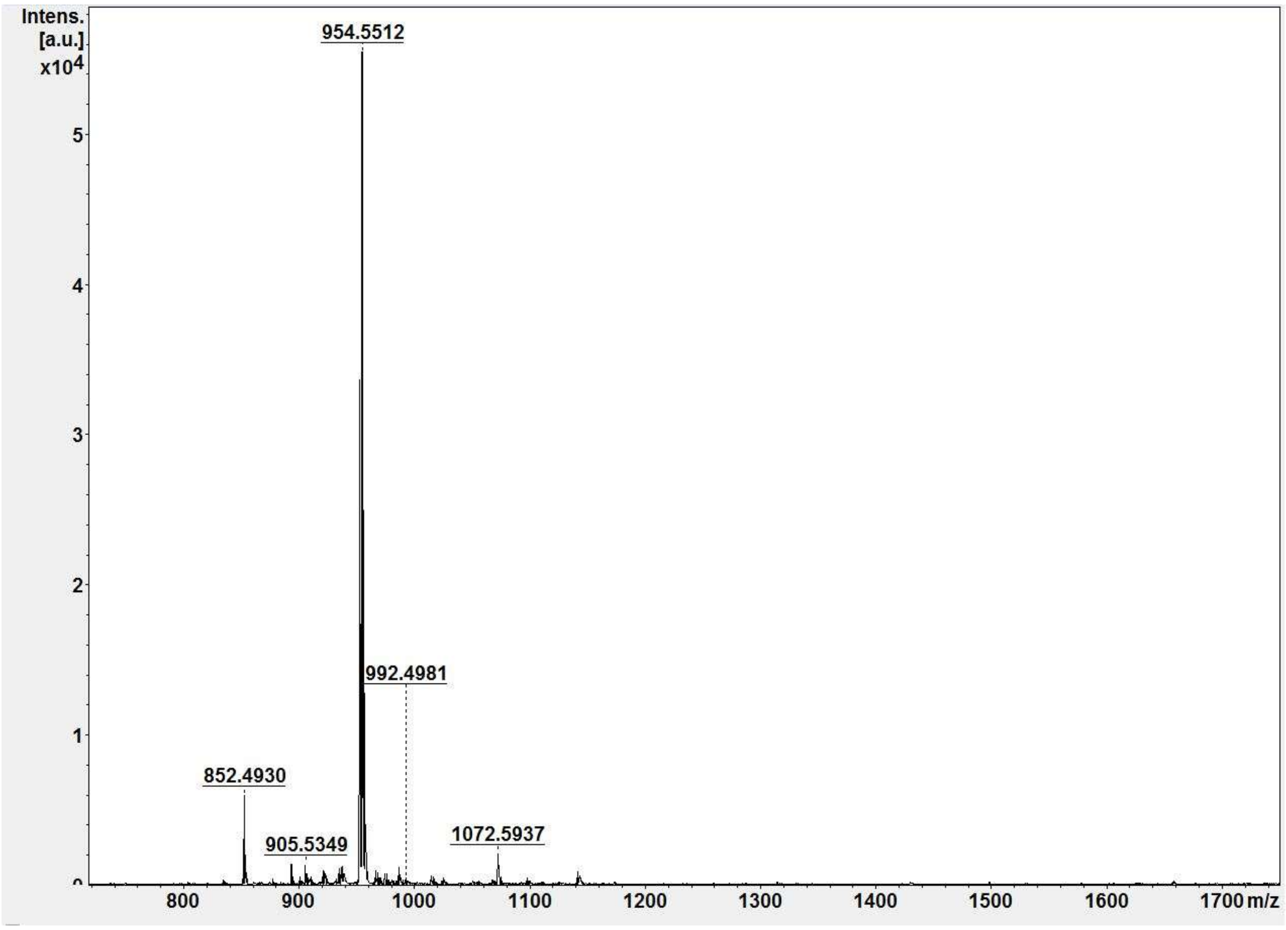

B: MALDI-TOF of peptide, **1** [CTVRTSADC] (Cyclized)

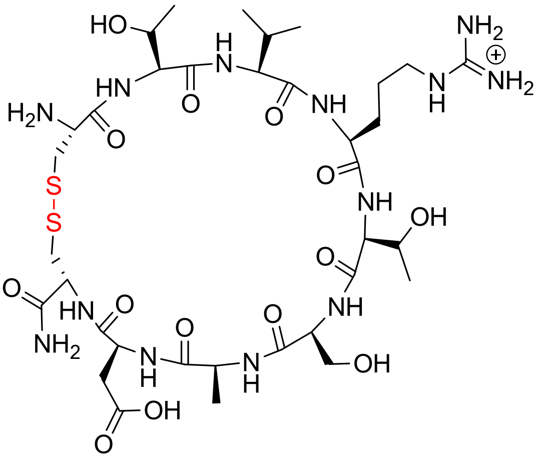

Exact Mass: 952.3975

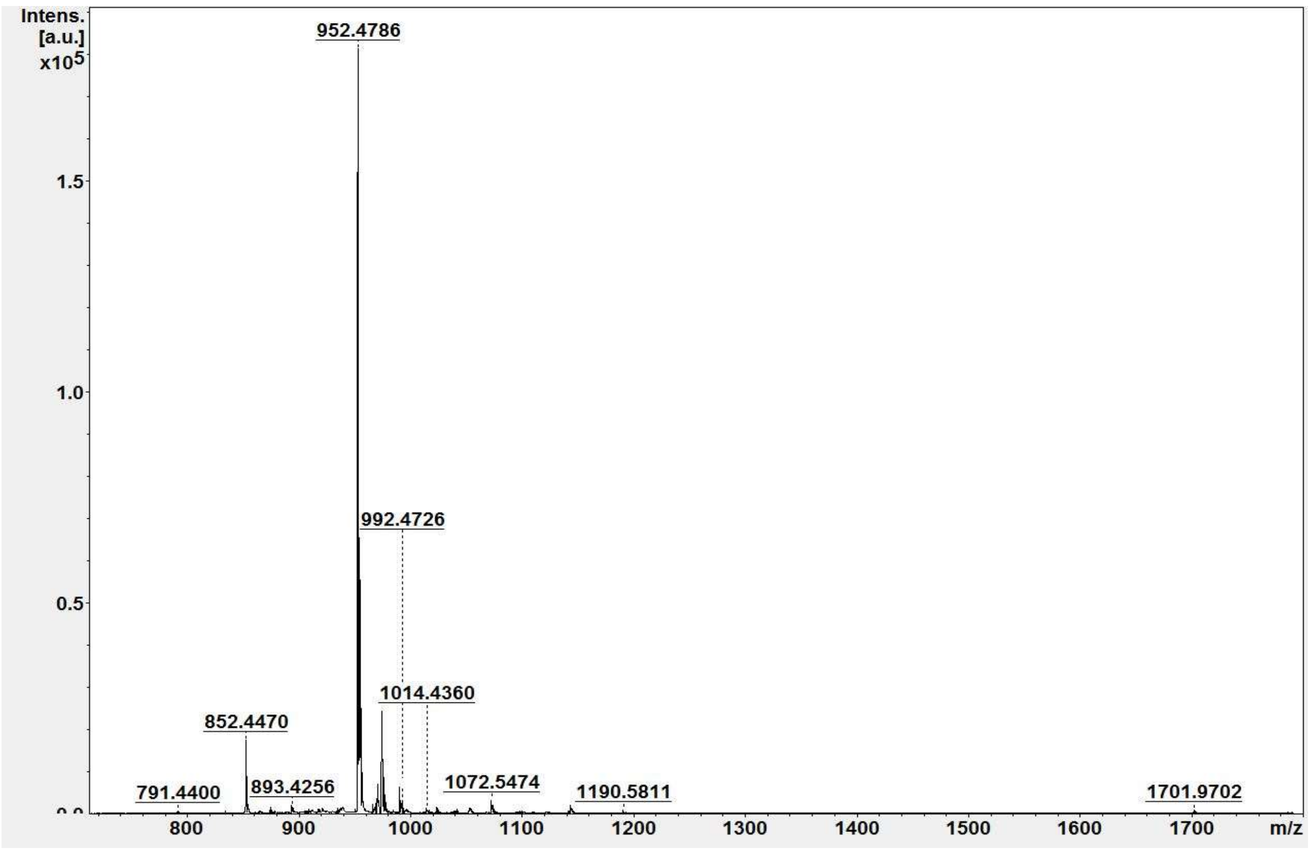

C: MALDI-TOF of intermediate peptide Fmoc-K(Dde)TVRTSADE

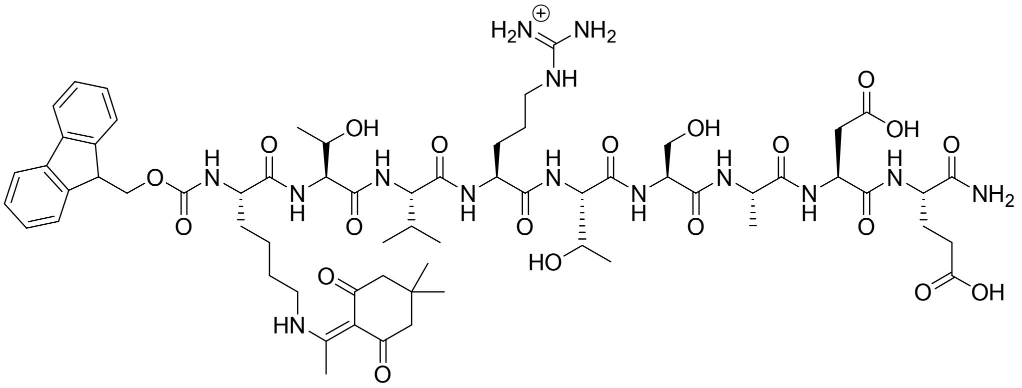

Exact Mass: 1391.6842

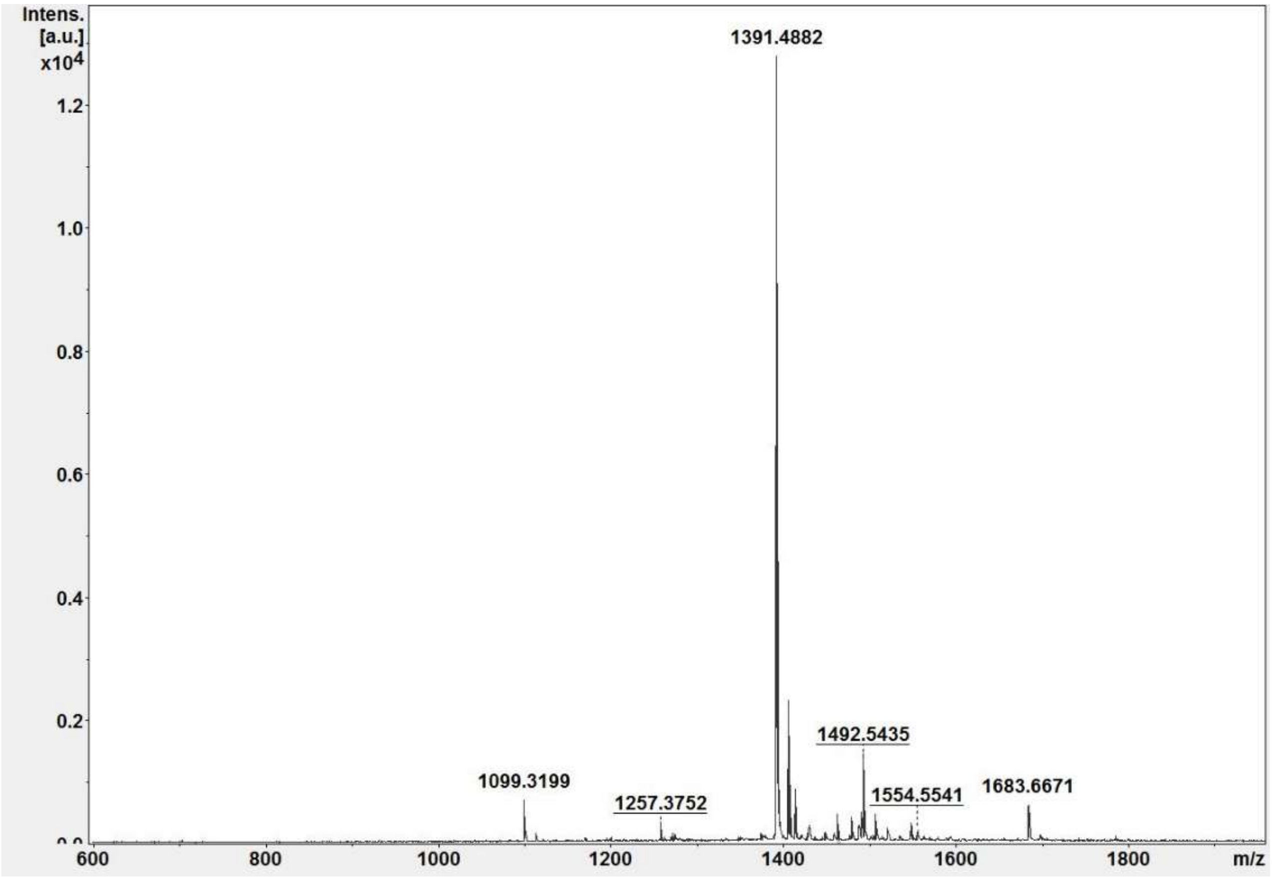

D: MALDI-TOF of intermediate peptide, Fmoc-KTVRTSADE

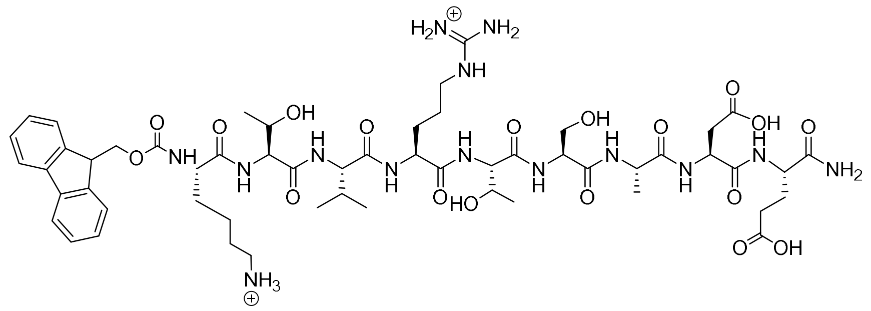

Exact Mass: 1228.6077

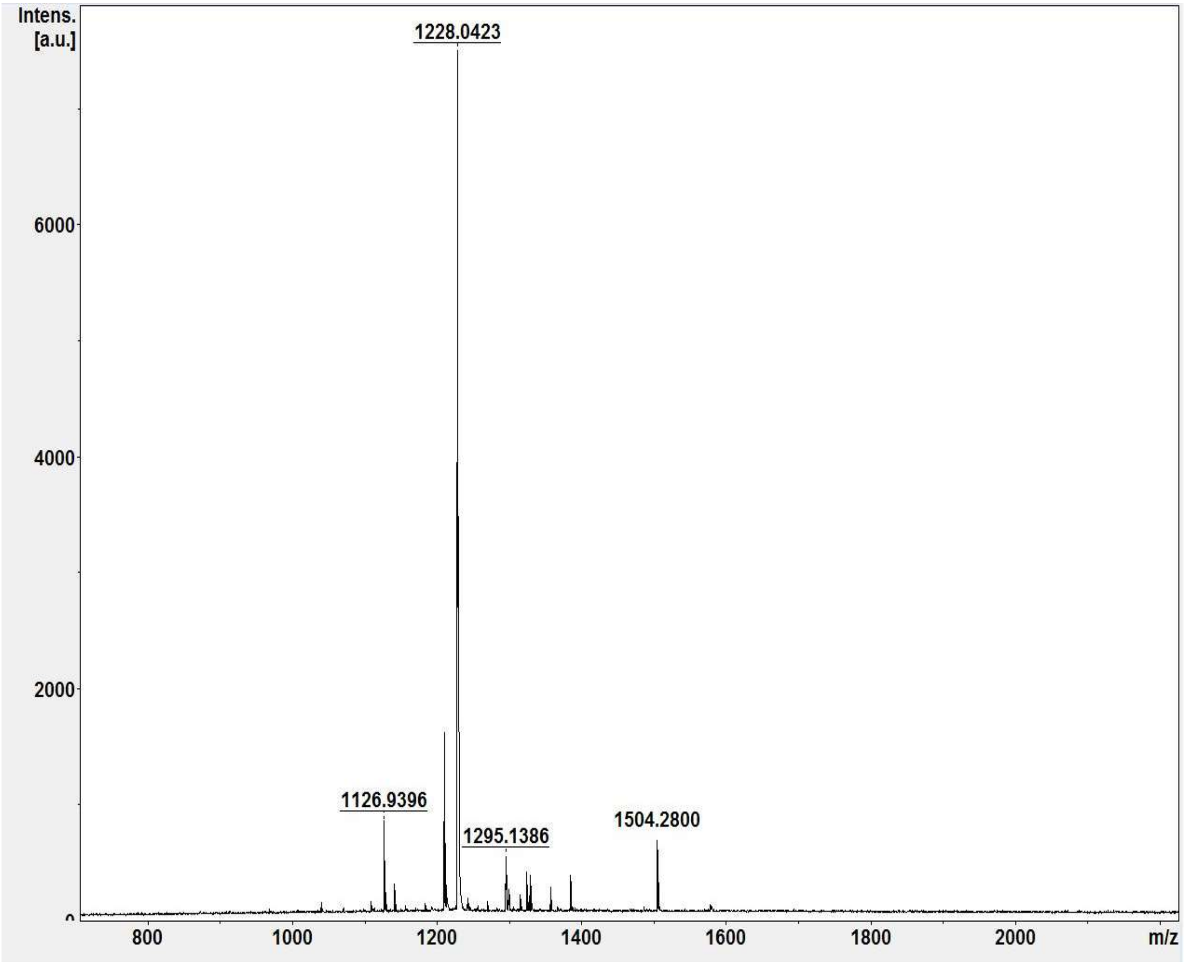

E: MALDI-TOF of peptide **8,** [KTVRTSADE] (cyclized)

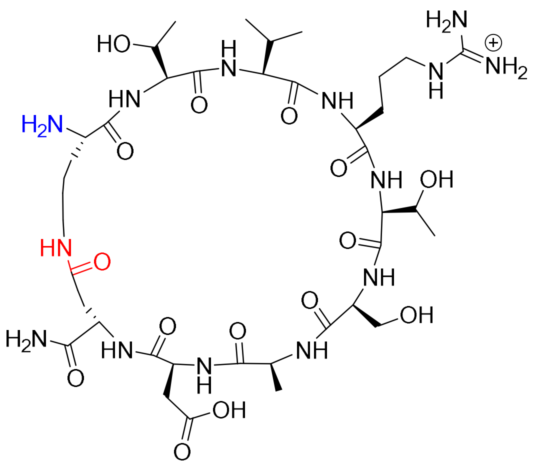

Exact Mass: 987.5218

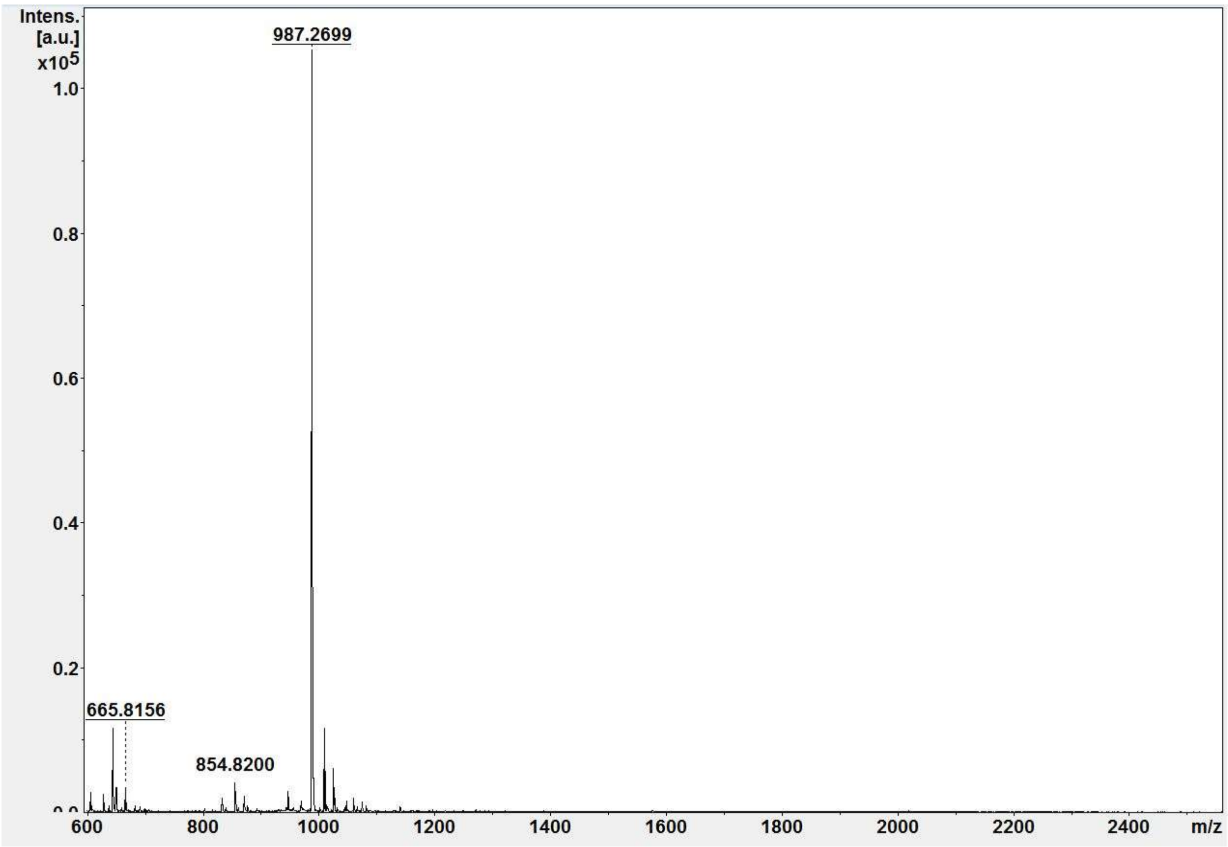

F: MALDI-TOF of peptide **5,** Hydrz-Glutrt-GG-FK-C_6-[CTVRTSADC]

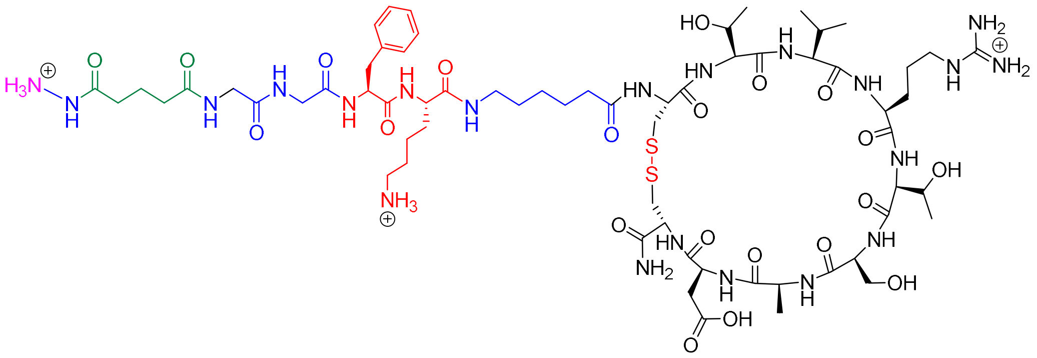

Exact Mass: 1584.7610

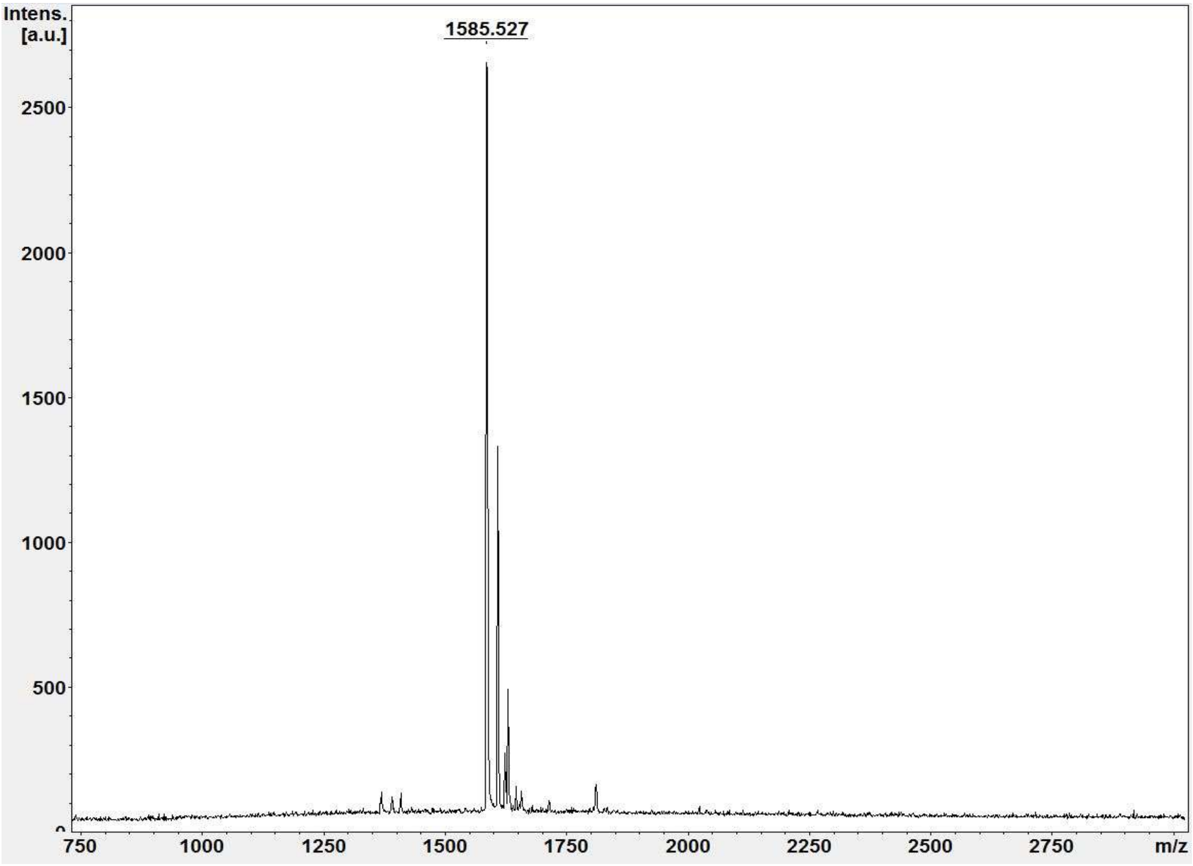

G: MALDI-TOF of peptide **6,** Hydrz-Glutrt-GG-VCit-C_6-[CTVRTSADC]

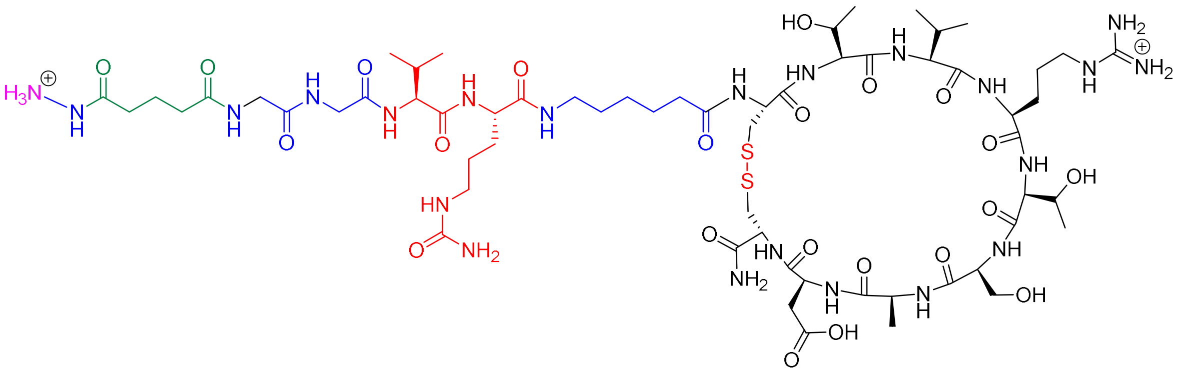

Exact Mass: 1564.7439

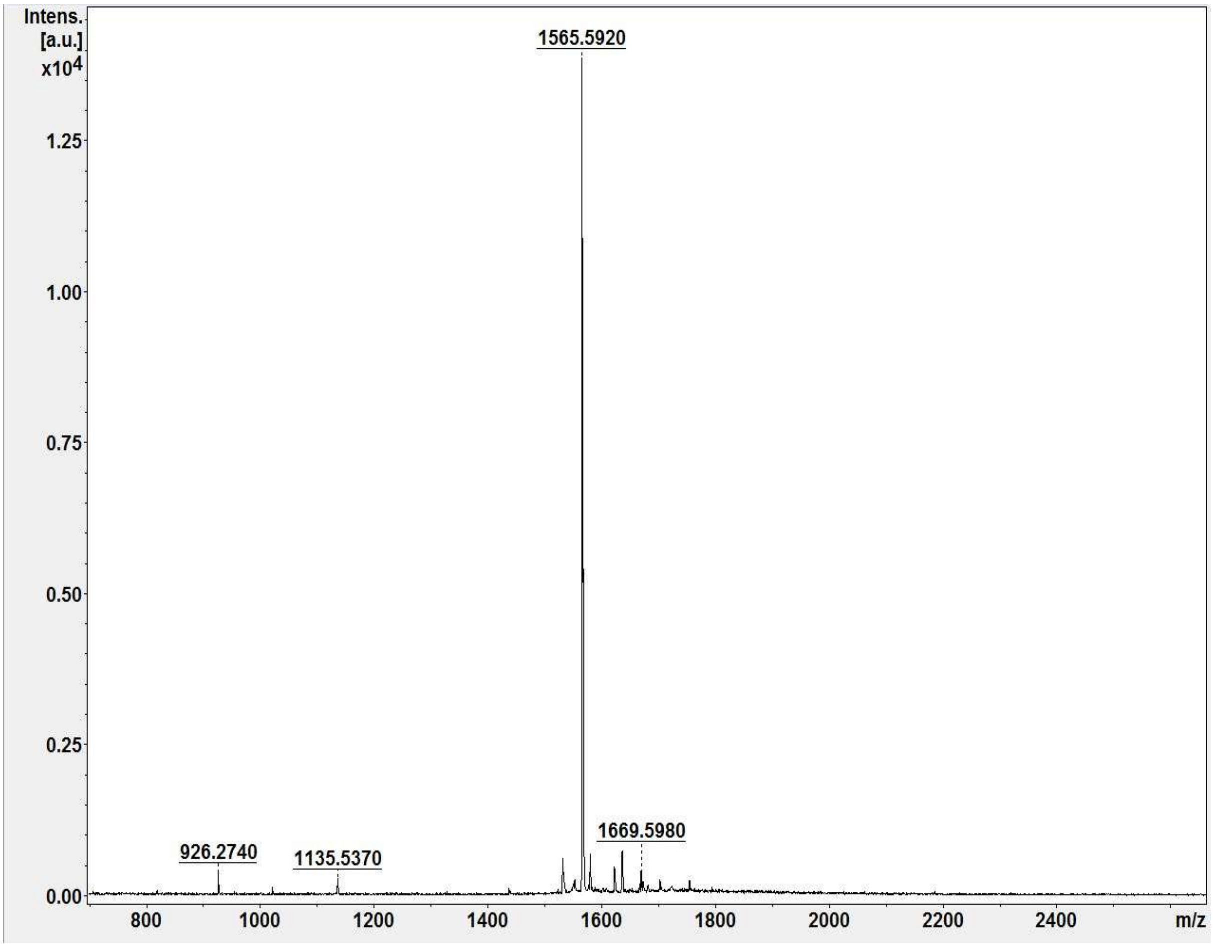

H: MALDI-TOF of peptide **9**, C-GFLC-C_6-[KTVRTSADE]

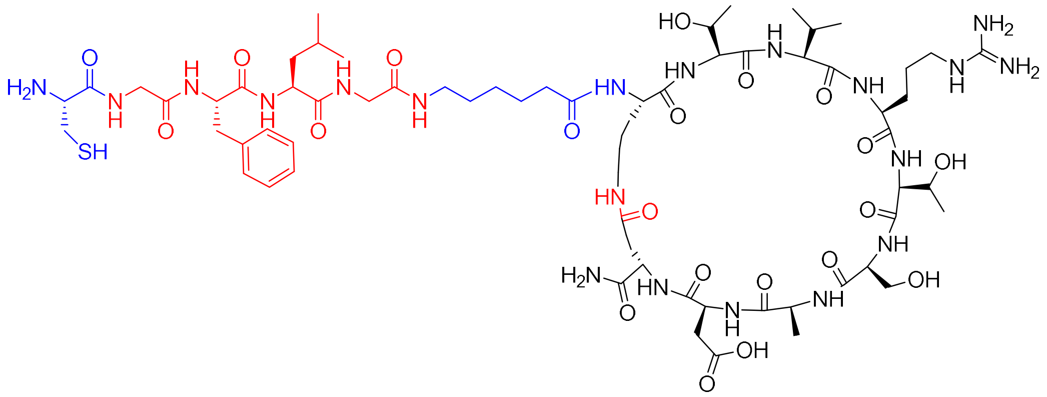

Exact Mass: 1577.8110

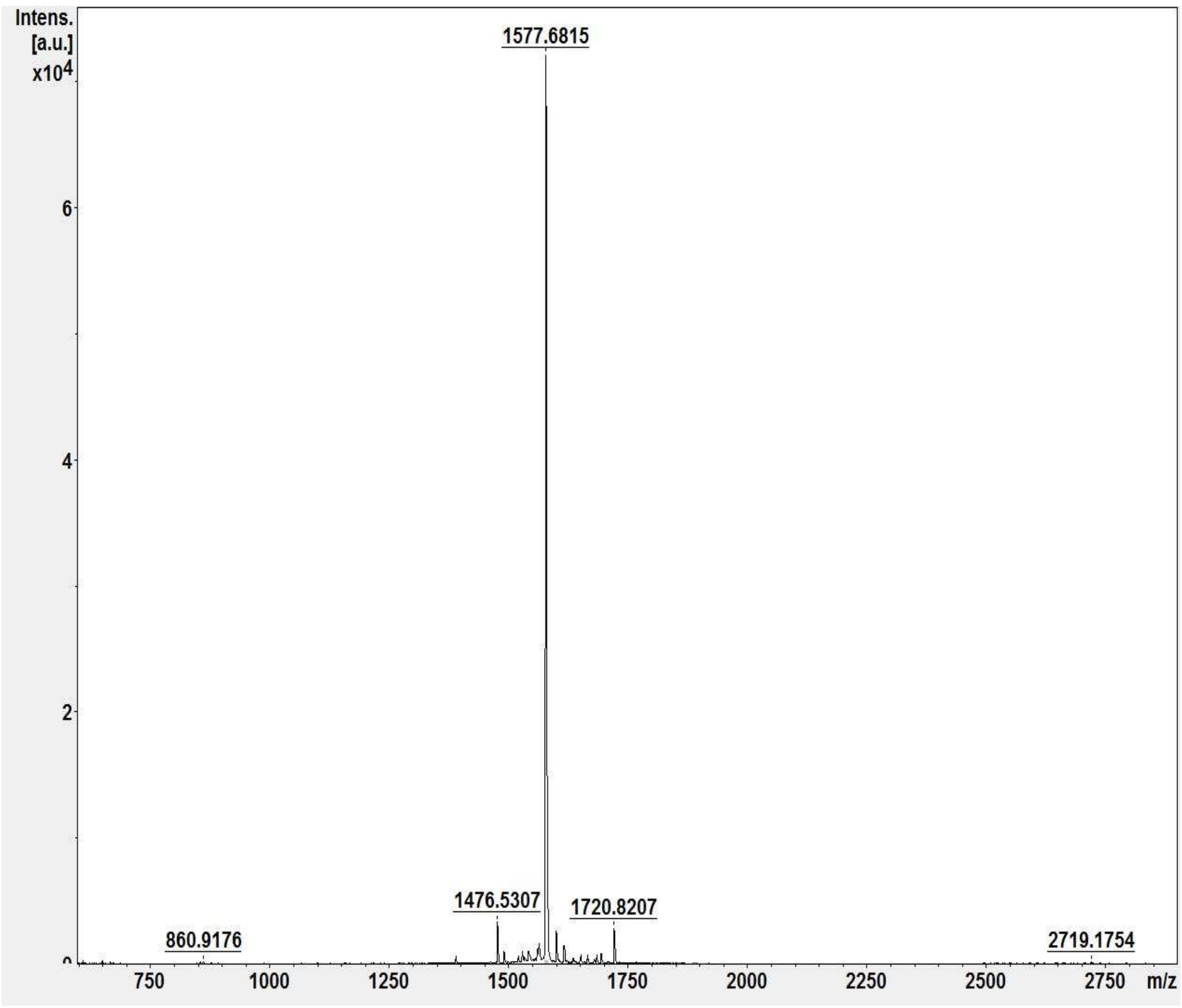

I: Analytical HPLC profile of peptide **9**, C-GFLC-C_6-[KTVRTSADE]

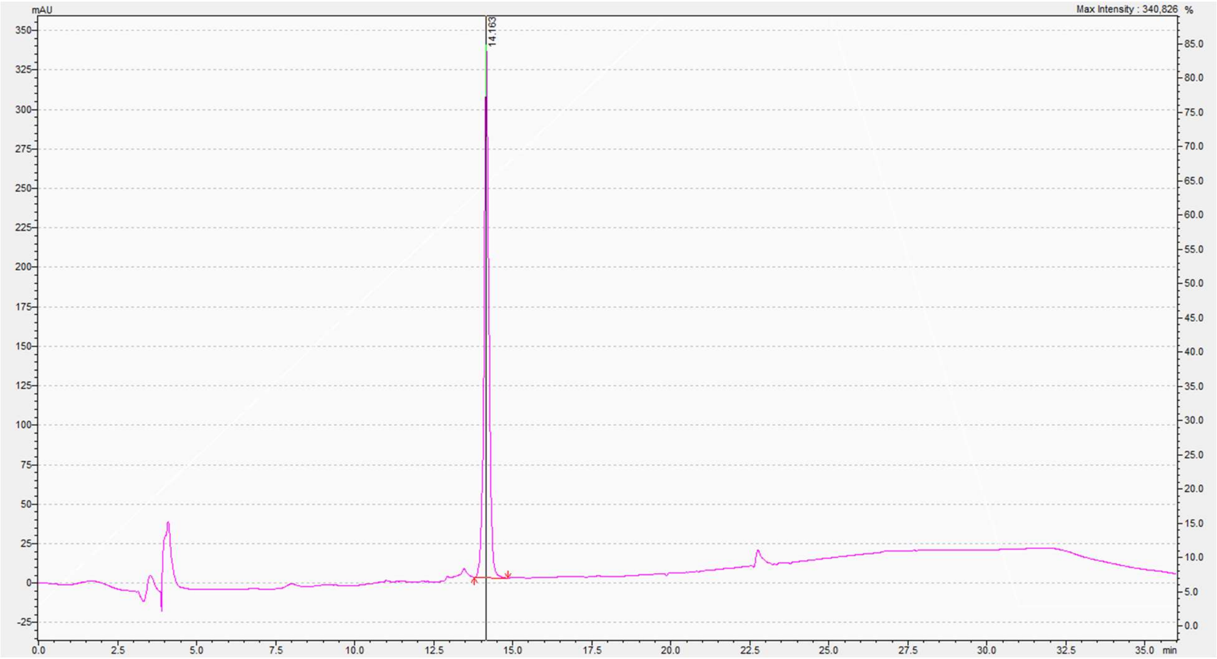

J: Analytical HPLC profile of compound **10**, Dox-s-s-pyridine

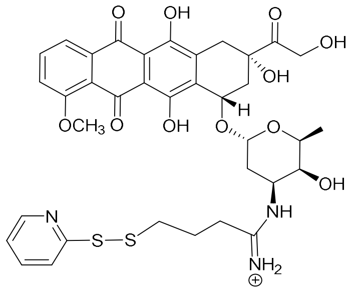

Exact Mass: 754.2099

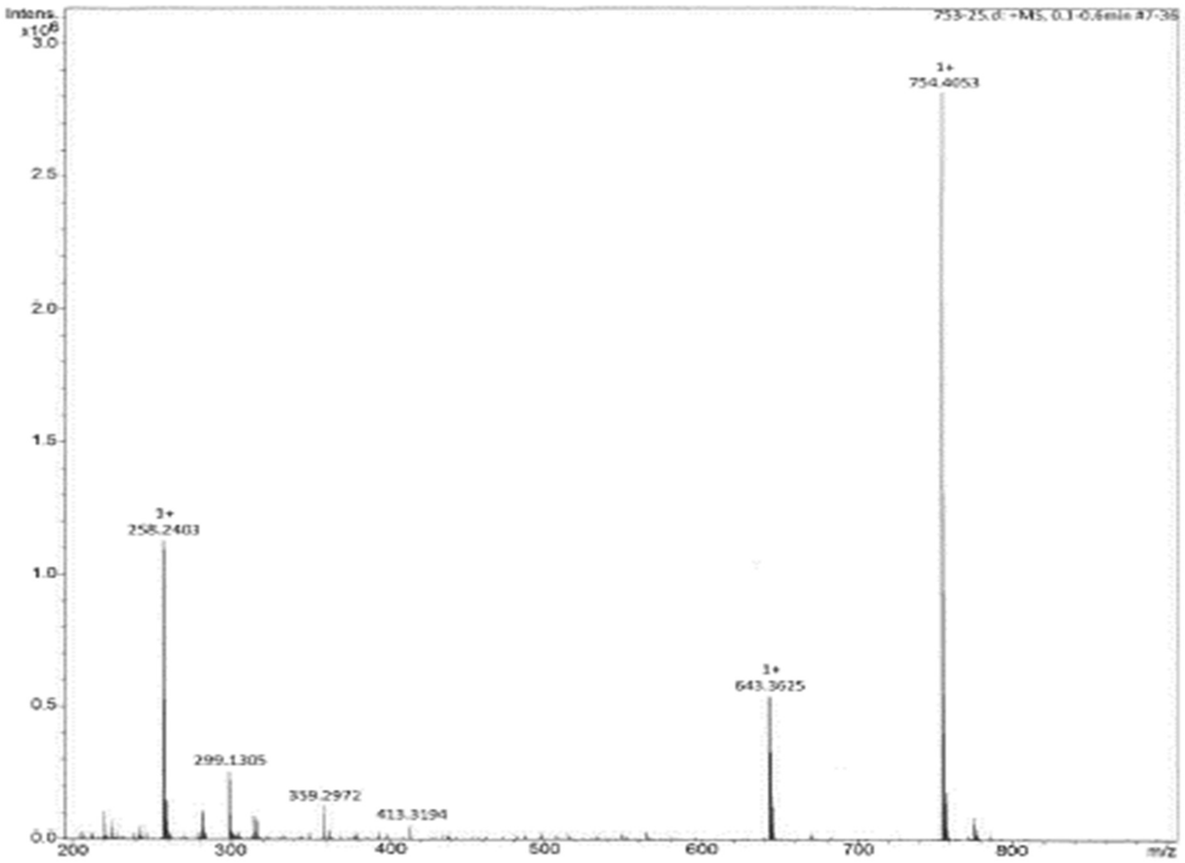

SHIMADZU LabSolutions

Analysis Report

<Sample Information>

Sample Name	: 753-25-1		
Sample ID	: 753-25-1		
Data Filename	: 753-25-2.lcd		
Method Filename	: 645-1.lcm		
Batch Filename	:		
Vial #	: 1-2	Sample Type	: Unknown
Injection Volume	: 10 uL		
Date Acquired	: 1/25/2017 7:42:11 AM	Acquired by	: DARWISH
Date Processed	: 1/25/2017 8:00:12 AM	Processed by	: DARWISH

<Chromatogram>

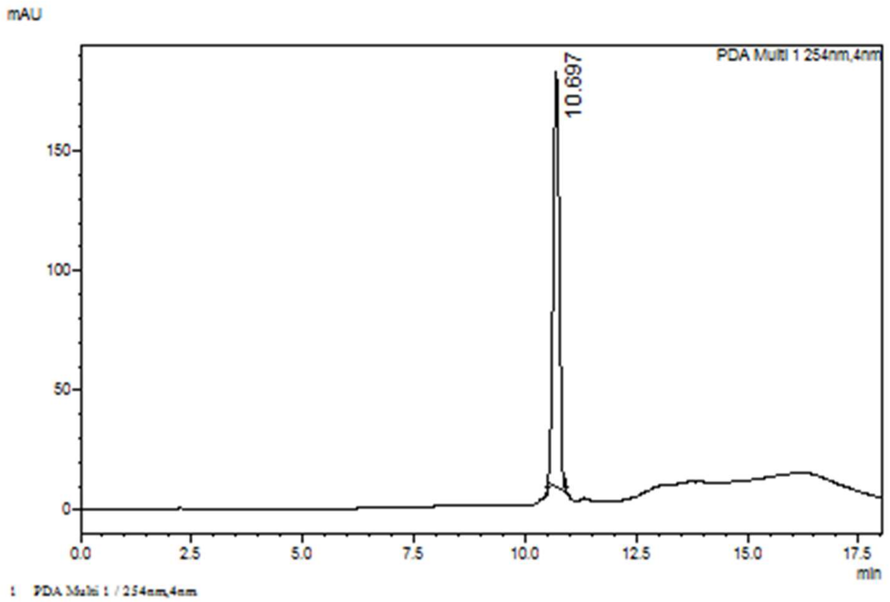

1 PDA Multi 1 / 254nm,4nm

K: MALDI-TOF of peptide conjugate**12**, Dox-hydrz-Glutrt–GG–VCit–C_6-[CTVRTSADC

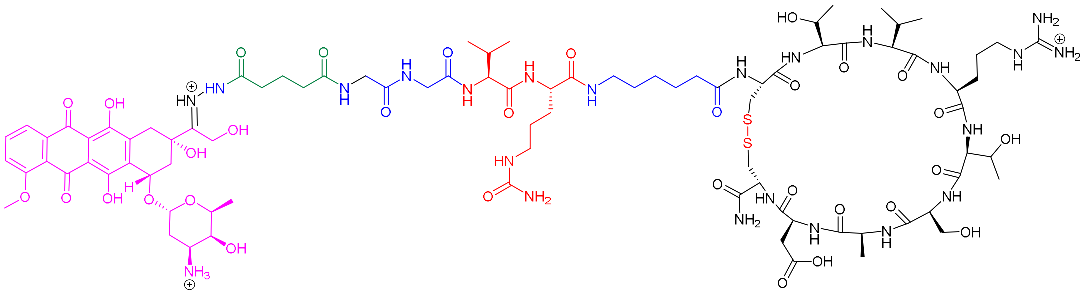

Exact Mass: 2090.9147

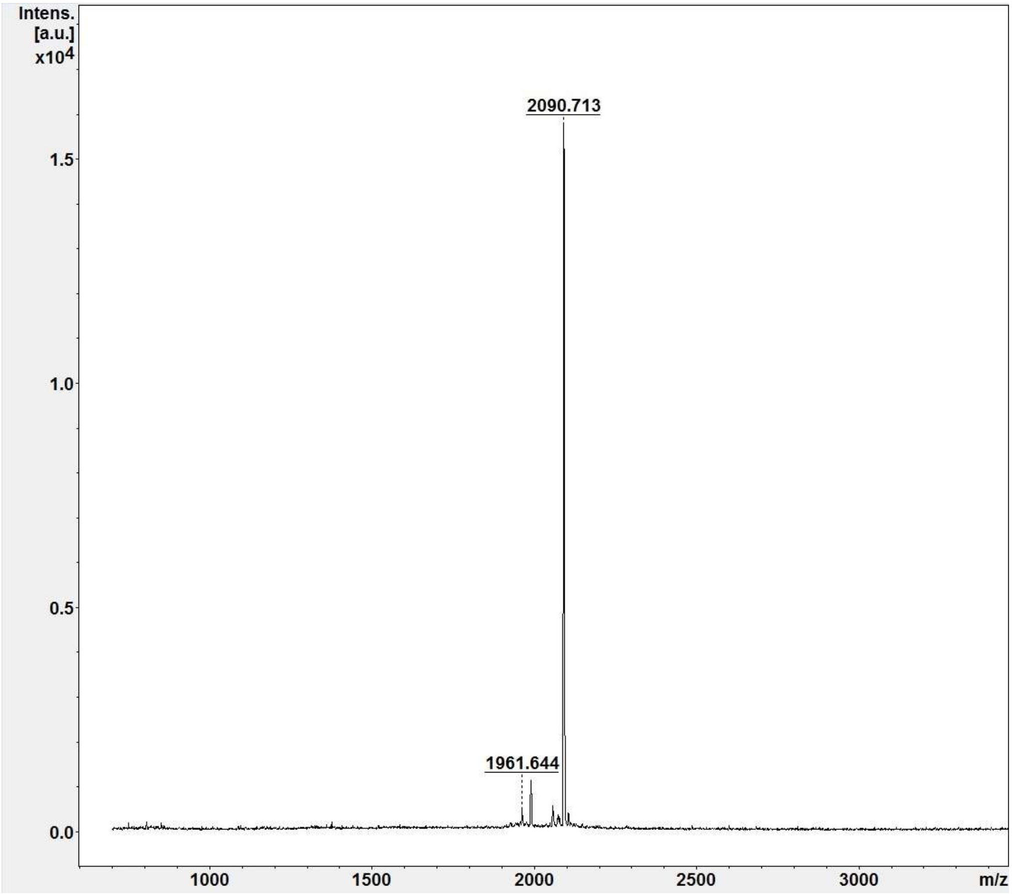

L: MALDI-TOF of peptide conjugate**13**, Dox-s-s-CGFLG-C_6-[KTVRTSADE]

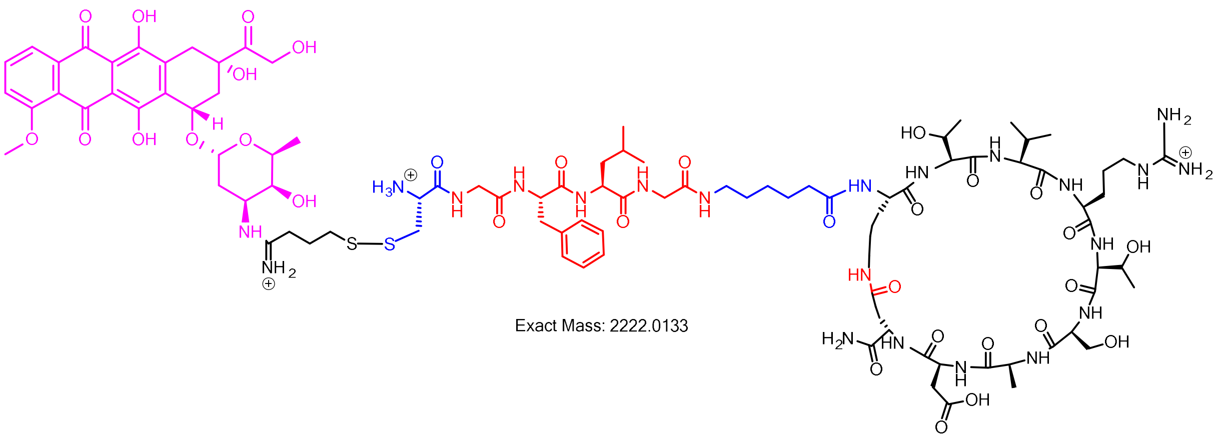

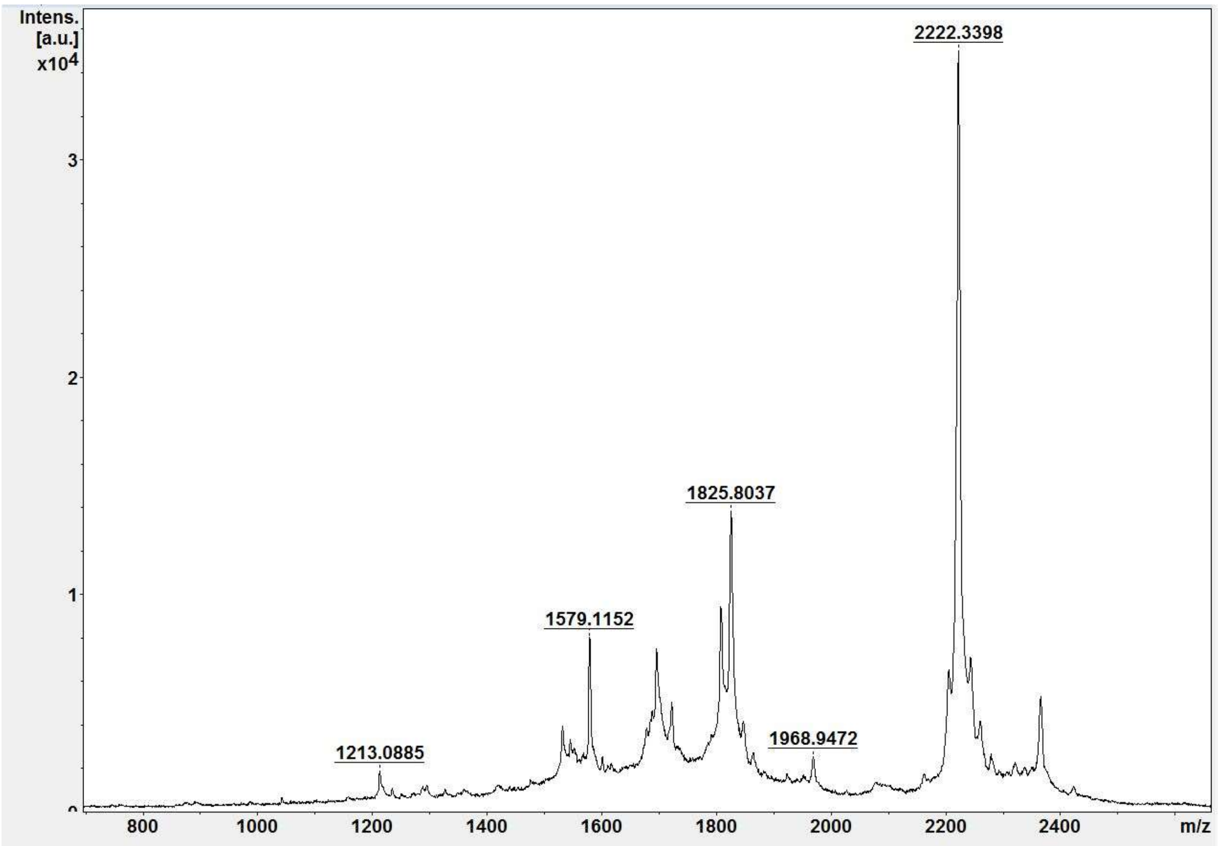

M: Analytical HPLC profile of peptide **13**, Dox-s-s-CGFLG-C_6-[KTVRTSADE]

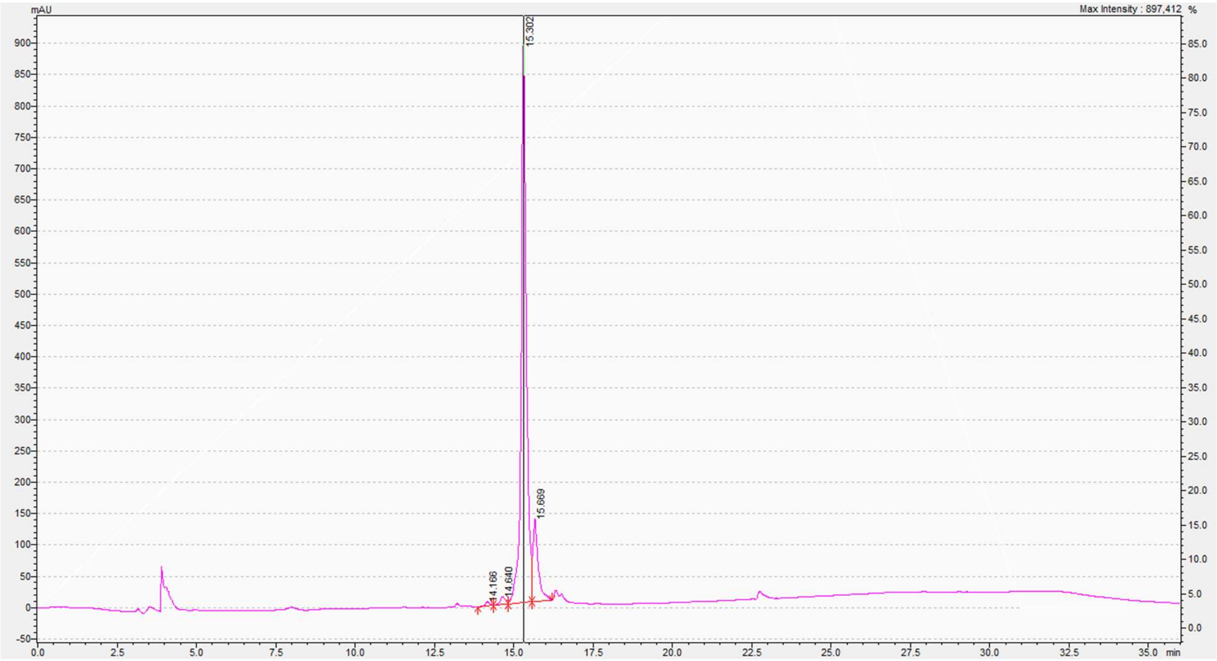

N: MALDI-TOF of peptide conjugate **14**, Doce-βA-thioether-CGFLG-C_6-[KTVRTSADE]

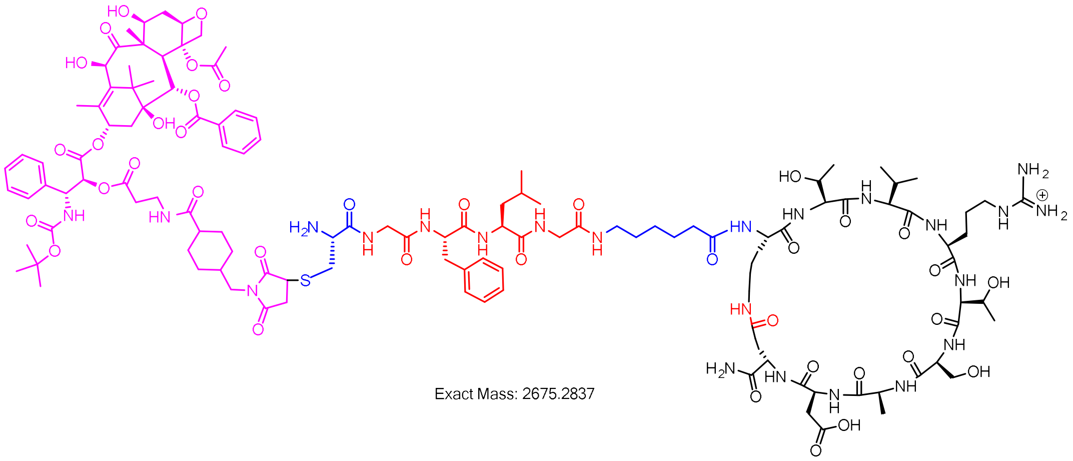

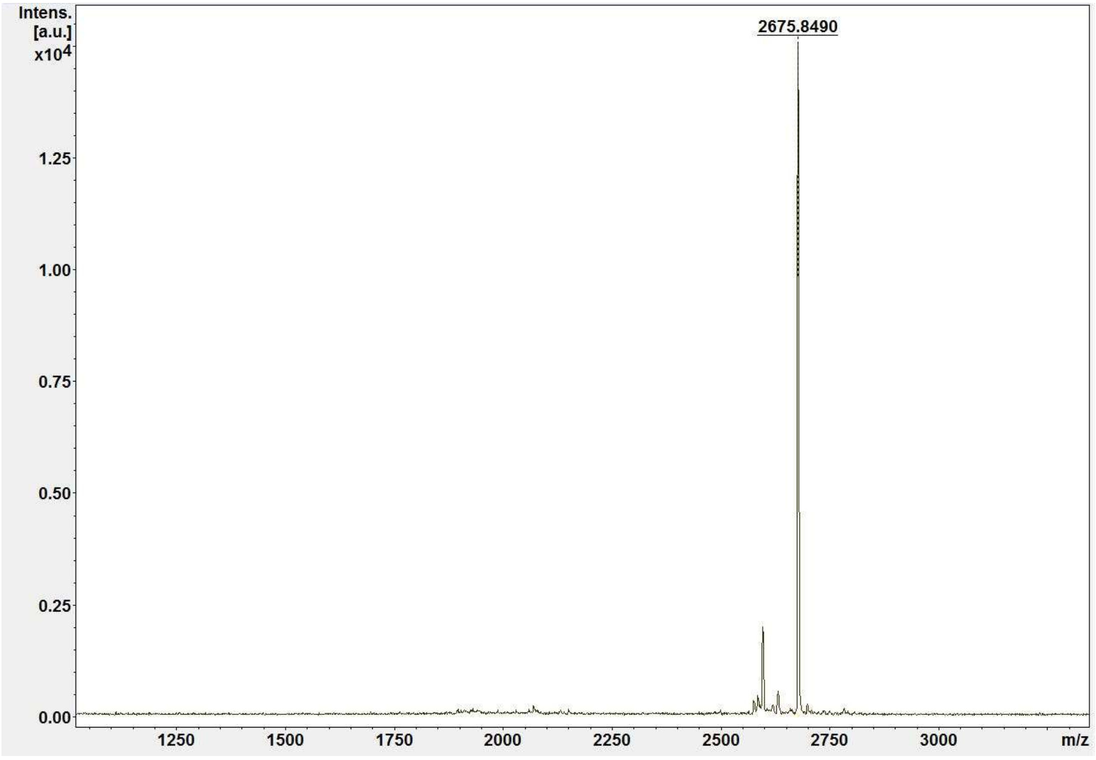

O: MALDI-TOF of peptide **17**, CFDI-GFLG-C_6-[CTVRTSADC]

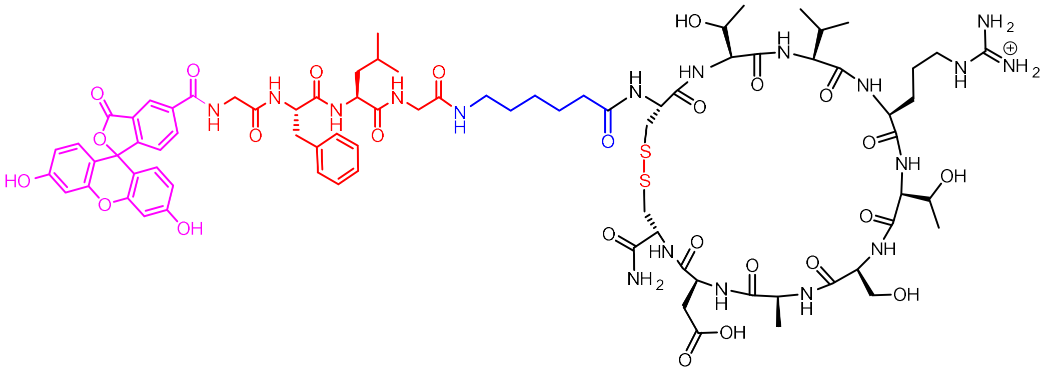

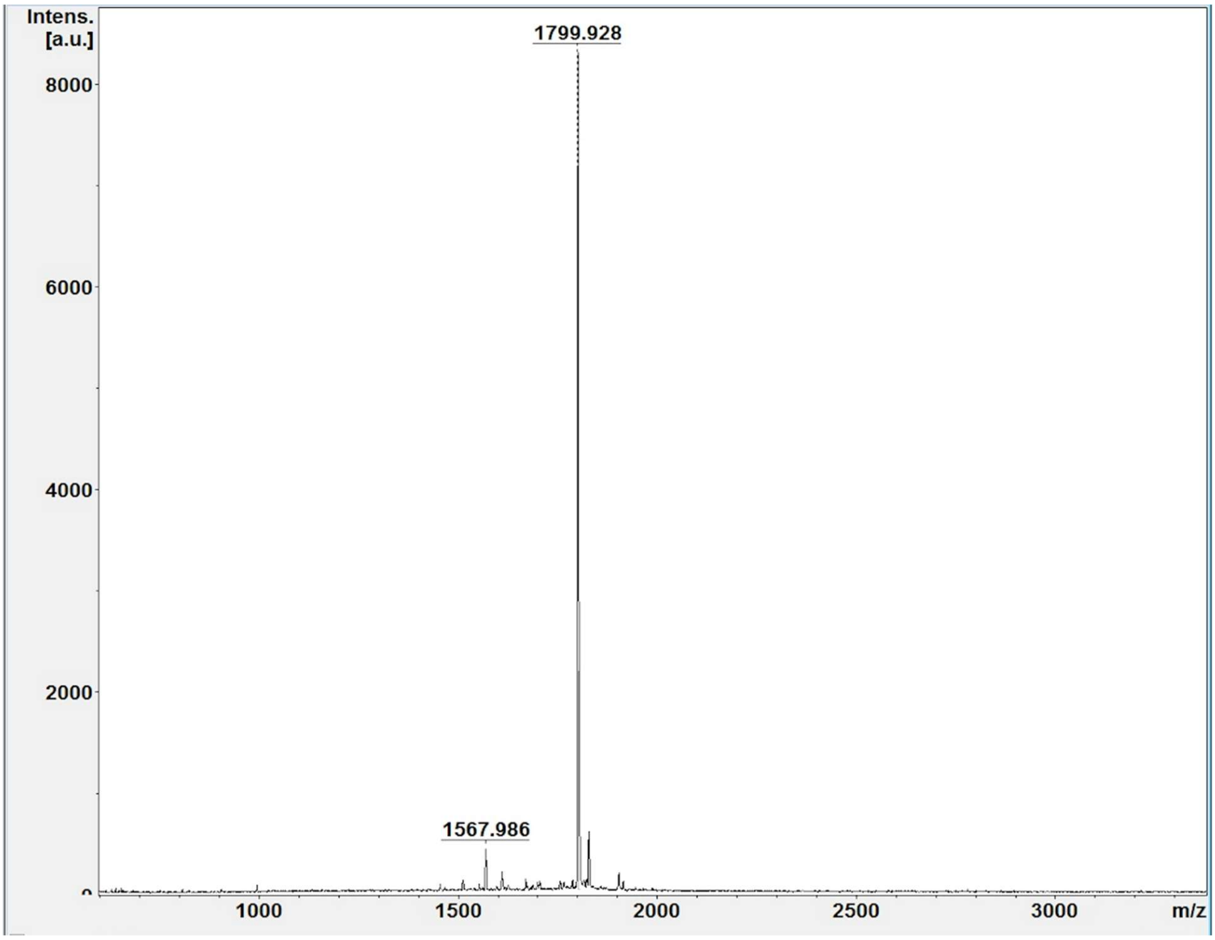

P: MALDI-TOF of peptide **18**, CFDI-GFLG-C_6-[KTVRTSADE]

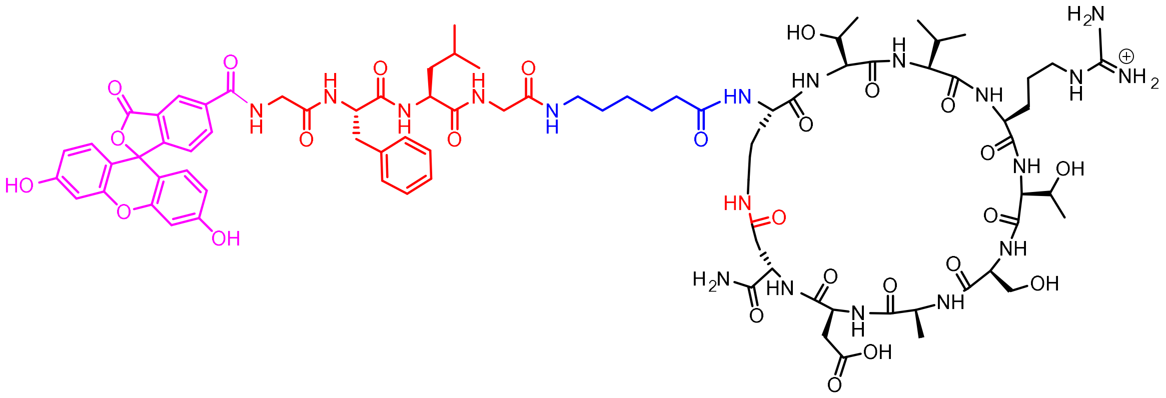

Exact Mass: 1832.8490

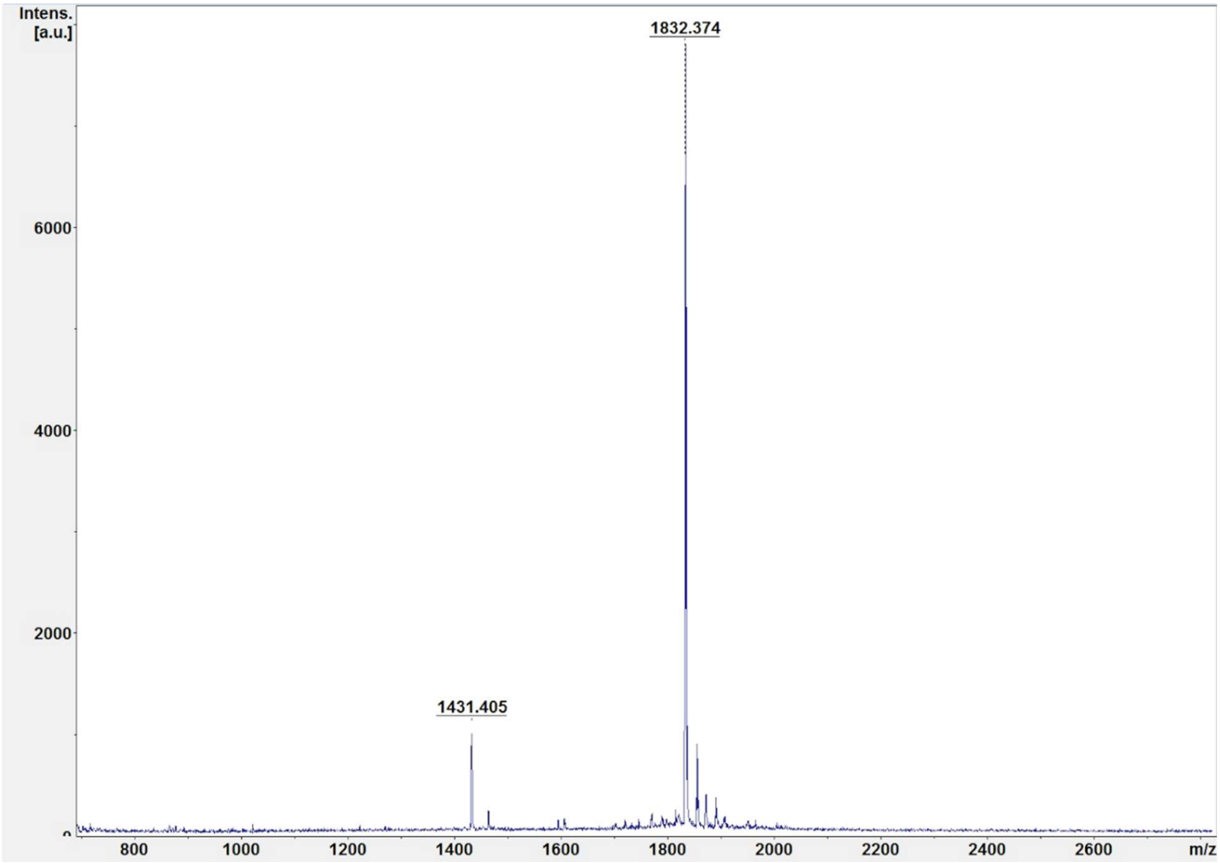

Q: MALDI-TOF of peptide **19**, C-GFLC-C_6-[CTVRTSADC

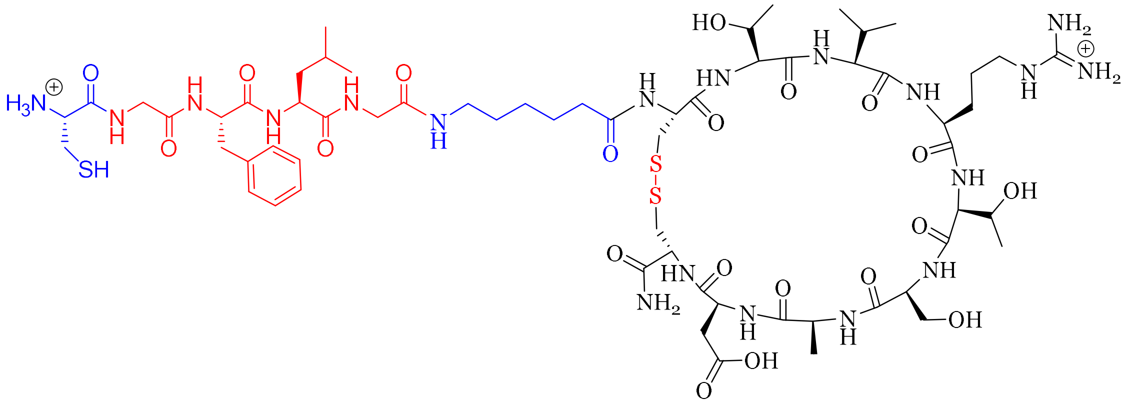

Exact Mass: 1543.6934

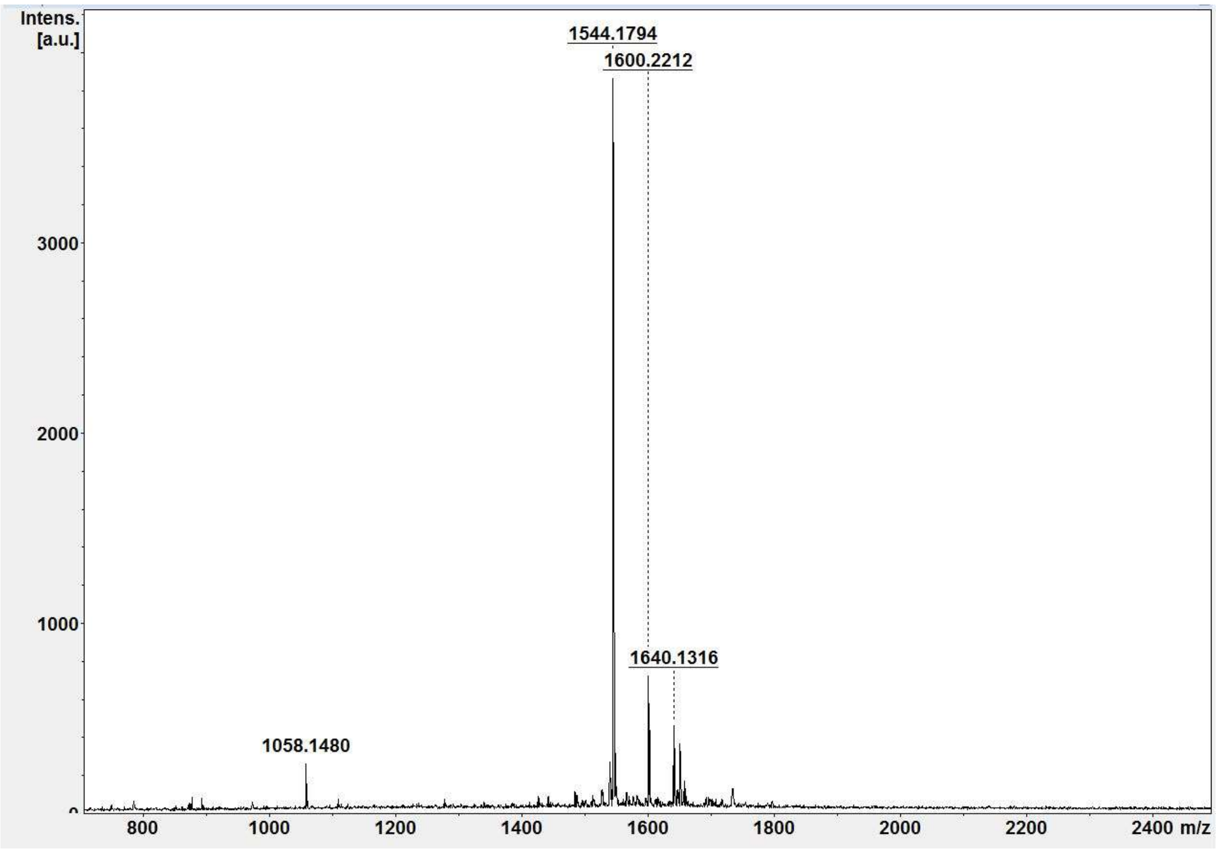

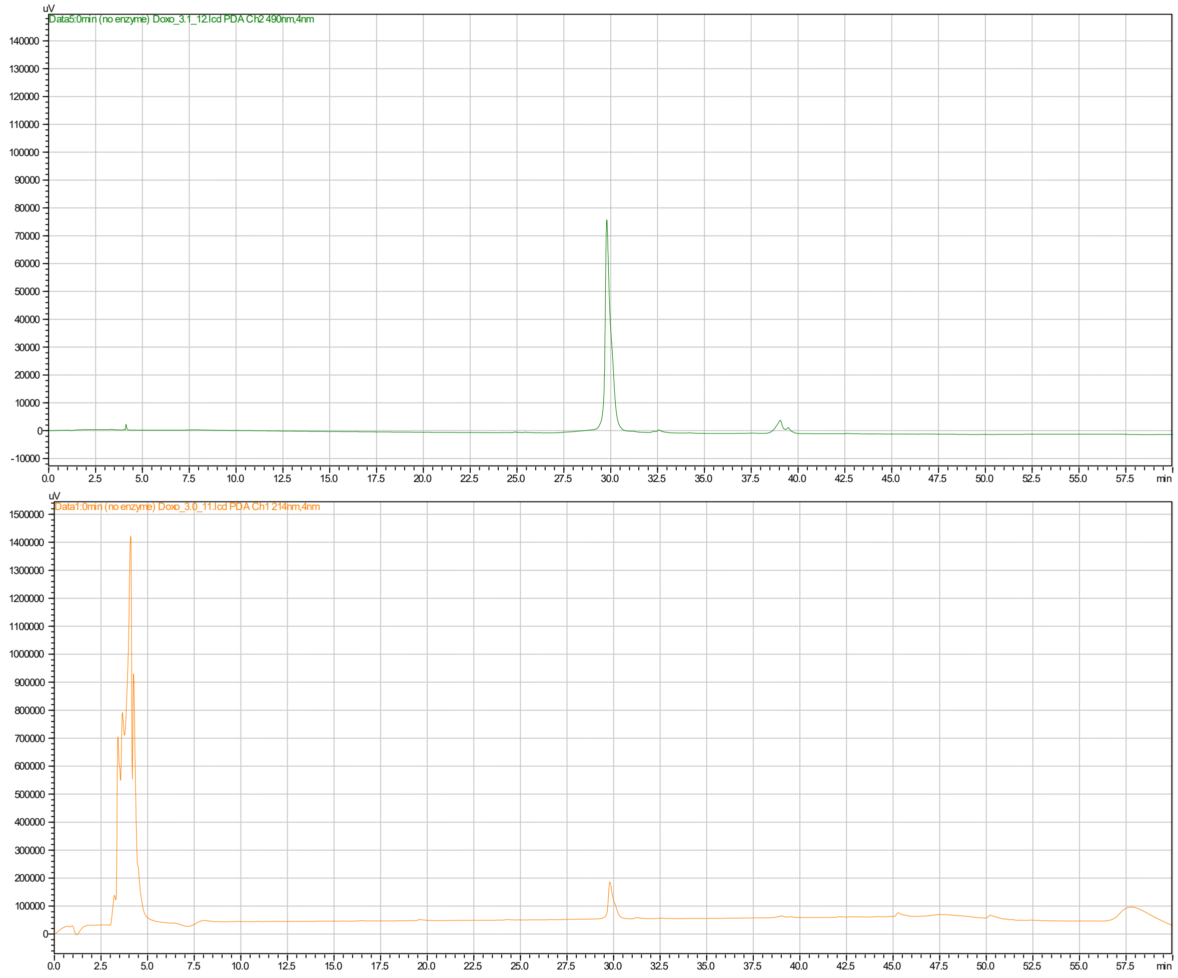

Figure S1. Analytical HPLC chromatogram for doxorubicin alone with the absorbance detection at 214 nm (top) and 490 nm (bottom).

(a)

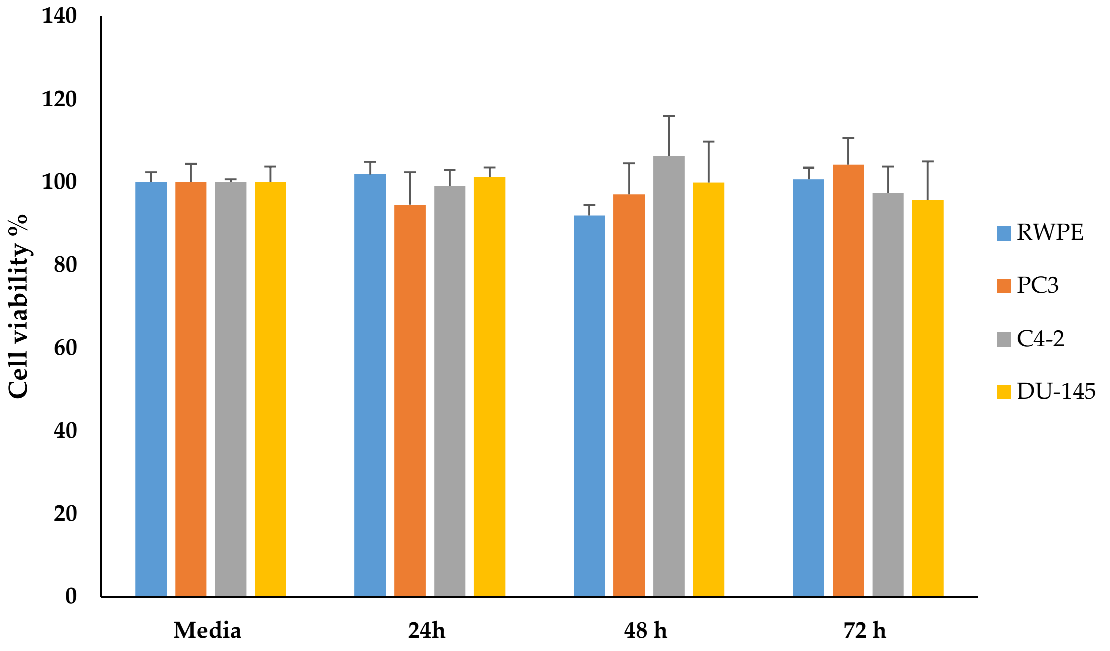
140
120
100
80
60
40
20
0
Cell viability %
Media
24h
48 h
72 h
RWPE
PC3
C4-2
DU-145

(b)

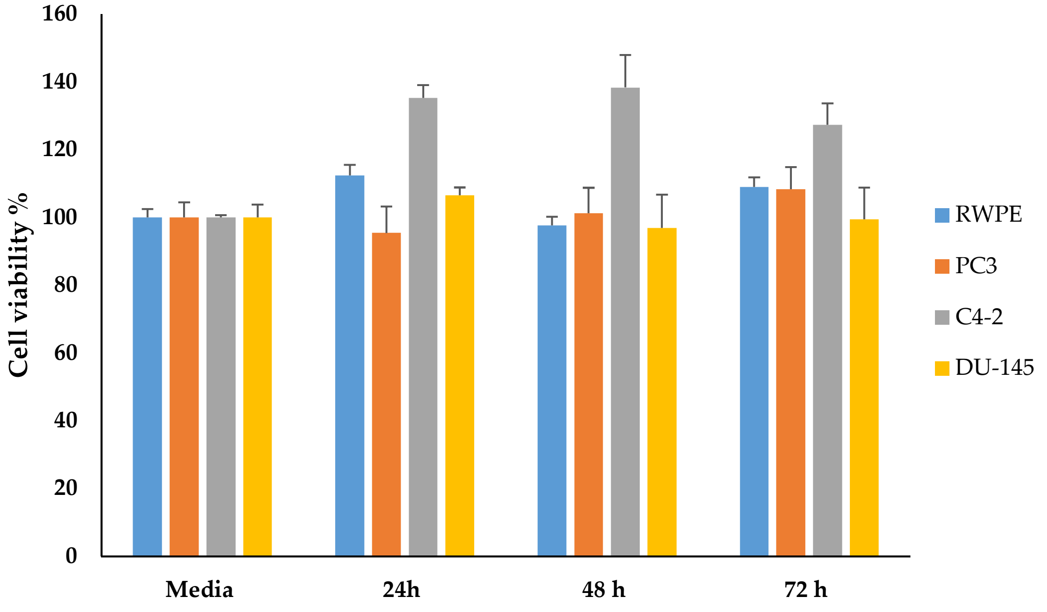
160
140
120
100
80
60
40
20
0
Cell viability %
Media
24h
48 h
72 h
RWPE
PC3
C4-2
DU-145

(c)

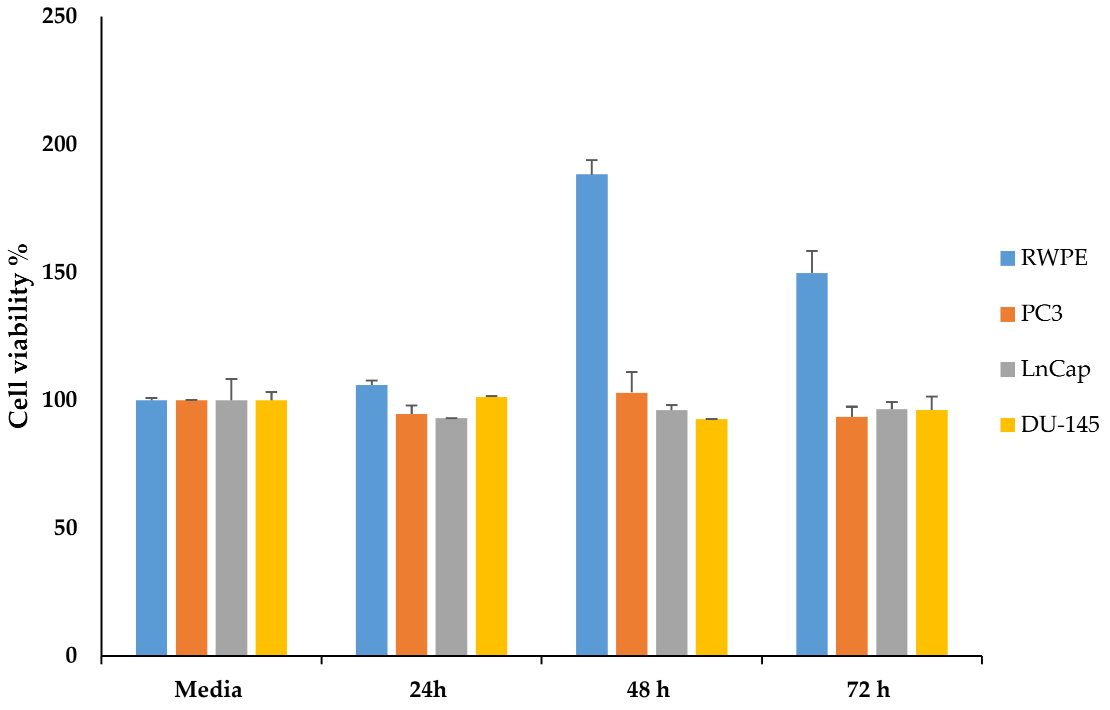

Figure S2. Cytotoxicity of a) peptide **5** (5 µM); b) peptide **6** (5 µM); c) peptide **9** (5 µM) after 24, 48, and 72 h incubation with RWPE-1, PC3, C4-2, and DU-145 cell lines.
